# Structural Insights into Curli CsgA Cross-β Fibril Architecture Inspire Repurposing of Anti-amyloid Compounds as Anti-biofilm Agents

**DOI:** 10.1371/journal.ppat.1007978

**Published:** 2019-08-30

**Authors:** Sergei Perov, Ofir Lidor, Nir Salinas, Nimrod Golan, Einav Tayeb- Fligelman, Maya Deshmukh, Dieter Willbold, Meytal Landau

**Affiliations:** 1 Department of Biology, Technion-Israel Institute of Technology, Haifa, Israel; 2 Institute of Complex Systems (ICS-6, Structural Biochemistry), Forschungszentrum Jülich, Jülich, Germany; 3 Institut für Physikalische Biologie, Heinrich-Heine-Universität Düsseldorf, Düsseldorf, Germany; UC Santa Cruz, UNITED STATES

## Abstract

Curli amyloid fibrils secreted by *Enterobacteriaceae* mediate host cell adhesion and contribute to biofilm formation, thereby promoting bacterial resistance to environmental stressors. Here, we present crystal structures of amyloid-forming segments from the major curli subunit, CsgA, revealing steric zipper fibrils of tightly mated β-sheets, demonstrating a structural link between curli and human pathological amyloids. D-enantiomeric peptides, originally developed to interfere with Alzheimer’s disease-associated amyloid-β, inhibited CsgA fibrillation and reduced biofilm formation in *Salmonella typhimurium*. Moreover, as previously shown, CsgA fibrils cross-seeded fibrillation of amyloid-β, providing support for the proposed structural resemblance and potential for cross-species amyloid interactions. The presented findings provide structural insights into amyloidogenic regions important for curli formation, suggest a novel strategy for disrupting amyloid-structured biofilms, and hypothesize on the formation of self-propagating prion-like species originating from a microbial source that could influence neurodegenerative diseases.

## Author summary

Atomic resolution structural insights into the biofilm-associated curli amyloid fibril secreted by *Enterobacteriaceae* revealed elements of fibrillar architecture conserved between bacterial and human amyloids. This inspired us to repurpose anti-amyloid drugs designed to target human pathological amyloids as a novel class of anti-biofilm agents. Moreover, the results provide a molecular basis for understanding interspecies cross-seeding of amyloids through the generation of prion-like agents by molecular mimicry. This raises concerns regarding human exposure to exogenous sources of amyloids, such as contaminated food and amyloid-secreting microbes. Overall, we provide a novel framework for investigating interspecies amyloid interactions at the molecular level and offer novel insights into mechanisms which may underlie the evolutionary and etiological relationships between the human and microbial amylomes.

## Introduction

Amyloid formation has traditionally been viewed as a hallmark of protein misfolding diseases, such as amyloidosis, Alzheimer’s and Parkinson’s [1]. In a groundbreaking study, Chapman and coworkers discovered that *Escherichia coli* secrete extracellular fibers called curli, that biochemically and biophysically resemble human pathological amyloids [2]. Curli proteins are produced via dedicated and tightly regulated cellular processes [3–5], and their fibrils mediate host cell adhesion and invasion, lead to immune system activation, and scaffold bacterial communities known as biofilms [6–9]. Functional amyloids have since been identified in all kingdoms of life, demonstrating a ubiquitous role for amyloids in physiological processes [2, 6, 9–13]. In microbes, the amyloids often serve as virulence determinants involved in aggressive infections and are thus attractive drug targets [9, 14].

Advances in structural biology methods have substantially contributed to the understanding of the remarkable stability of eukaryotic amyloids, ascribed to their shared cross-β structural feature, composed of tightly mated β-sheets with β-strands situated perpendicular to the fibril axis [15–26]. In contrast, the study of bacterial amyloids has proceeded in a long-standing vacuum of high-resolution structural knowledge. We have previously determined the first crystal structure of a bacterial amyloid fibril, i.e., the phenol soluble modulin α3 (PSMα3) peptide secreted by the pathogenic *Staphylococcus aureus* [27]. The structure revealed a fundamental polymorphism of the amyloid fold, showing that α-helices can replace the β-strands stacked perpendicular to the fibril axis, forming cross-α fibrils [27]. Fibrillation was shown to facilitate PSMα3 cytotoxicity against human cells [27, 28], supporting its classification as a functional bacterial amyloid involved in pathogenicity [27–29]. Furthermore, the secondary structure polymorphism of the PSMα3 cross-α fibrils was particularly striking and indicative of structurally-encoded functional specificity, considering that homologous family members, PSMα1 and PSMα4, form the canonical cross-β amyloid [30]. These PSMs are involved in *S*. *aureus* biofilm structuring, and their fibrils, composed of β-sheets mated through tight steric zipper interfaces [30], likely contribute to the stability of the biofilm matrix [31]. In addition, recent structural work in *Bacillus subtilis* live biofilms showed a swift structural change in TasA, another suspected amyloid protein, from a globular shape toward β-sheet–rich fibrils [32].

The two key players in curli fibril formation are CsgA and CsgB, the major and minor curli subunits, respectively [5]. CsgB nucleates CsgA fibrillation in-vivo via interactions with soluble and unstructured CsgA monomers secreted to the outer bacterial membrane [3–5]. This specific nucleation process likely ensures fibril homogeneity and integrity [33]. CsgA, a 151-residue protein, consists of five imperfect sequence repeats (R1-R5) (S1 Table), defined by regularly spaced serine (Ser), glutamine (Gln) and asparagine (Asn) residues. The first and the last repeats (R1 and R5) form amyloid fibrils in isolation and are critical to CsgA seeding and nucleation by CsgB [34], while the other repeats (R2-R4) contain 'gatekeeper' residues that temper the amyloidogenicity of CsgA [35–37]. CsgA fibrils are resistant to chemical and proteolytic degradation [2, 35, 38, 39], bind the amyloid indicator dyes thioflavin T (ThT) [40] and congo red (CR) and can be visualized, via transmission electron microscopy (TEM), surrounding the bacteria [2, 37, 41]. The X-ray fibril diffraction pattern of CsgA shows reflections at 4.6–4.7 Å and 8–10 Å, indicating a structural spacing typical of cross-β architecture [39, 41] reminiscent of human pathological amyloids. Solid-state NMR data have previously suggested that recombinant CsgA adopts the same structure as native curli isolated from the bacteria, validating much of the biophysical information obtained from recombinant CsgA [42].

Molecular structures of curli-associated proteins are needed to better understand how curli biogenesis is controlled. Structures are available for few curli accessory components, including solution NMR structures of the periplasmic accessory protein CsgE [43] and the adaptor protein CsgF [44], as well as crystal structures of the periplasmic chaperone CsgC [45], which inhibits intracellular CsgA fibrillation [4, 46], and the outer membrane secretion channel CsgG [47, 48]. Despite attempts to obtain structural information on curli fibrils, atomic resolution structures of CsgA and CsgB have remained elusive due to the limitations of some structural methods in contending with the intrinsic properties of full-length amyloid fibrils, which are insoluble and often polymorphic and partially disordered in nature [19, 25, 42, 49]. We thus adopted the reductionist approach of looking for amyloidogenic spine segments which are suggested to nucleate and form the structured backbone of amyloid fibrils [25], and explored segments of CsgA.

We revealed structural similarity between fibrillar spine segments derived from CsgA and those derived from human pathological amyloids, which prompted us to investigate whether fibrillation inhibitors designed against human amyloids could also inhibit curli formation. Accordingly, we found that two D-enantiomeric peptides, originally designed to interfere with the formation of oligomers of Alzheimer’s disease-associated amyloid-β (Aβ) [50–60], inhibited the fibrillation of CsgA spines as well as of full-length CsgA, and reduced biofilm formation in curli-expressing *Salmonella typhimurium*. Furthermore, in accordance with the structural similarity, fibril seeds of CsgA and of its amyloidogenic segments facilitated fibrillation of Aβ. The results provide structural insights into a biofilm-related amyloid, which could have bearing on amyloid diseases by cross-seeding and the creation of transmissible agents [61], as well as pave the way for the rational development of anti-microbial drugs targeting amyloid-structured biofilms.

## Results

### Atomic resolution structures of CsgA spine segments reveal canonical steric zippers characteristic of pathological amyloids

To investigate structural features of CsgA, we identified potential amyloid-forming segments that may function as structured spines of CsgA fibrils. These segments were selected by combining computational data predicting regions of amyloidogenic propensity [62–66]. We focused on _45_LNIYQY_50_ and _47_IYQYGG_52_ from the R1 repeat, _137_VTQVGF_142_ from the R5 repeat, and _129_TASNSS_134_ from the R4-R5 loop (sequence positions are indicated as subscript based on the sequence of CsgA with a Uniprot accession number P28307). _129_TASNSS_134_ was selected as a control sequence as it was computationally predicted to be amyloidogenic but is located in a region not implicated in fibrillation [34]. TEM micrographs demonstrated that all four segments formed fibrils (S1A Fig), in accordance with the predictions of their amyloidogenic propensities. However, while _45_LNIYQY_50_ (R1), _47_IYQYGG_52_ (R1), and _137_VTQVGF_142_ (R5) formed unbranched and elongated amyloid-like fibrils, the _129_TASNSS_134_ (R4-R5 loop) segment formed scarce and more amorphous structures (S1A Fig). The _45_LNIYQY_50_ (R1), _47_IYQYGG_52_ (R1), and _137_VTQVGF_142_ (R5) segments bound the amyloid indicator dye ThT and demonstrated dose-dependent amyloid fibrillation curves with short lag times (S2 Fig). _45_LNIYQY_50_ polymerized with the shortest lag time. The _129_TASNSS_134_ segment from the R4-R5 loop did not bind ThT (S2 Fig).

**Fig 1:**
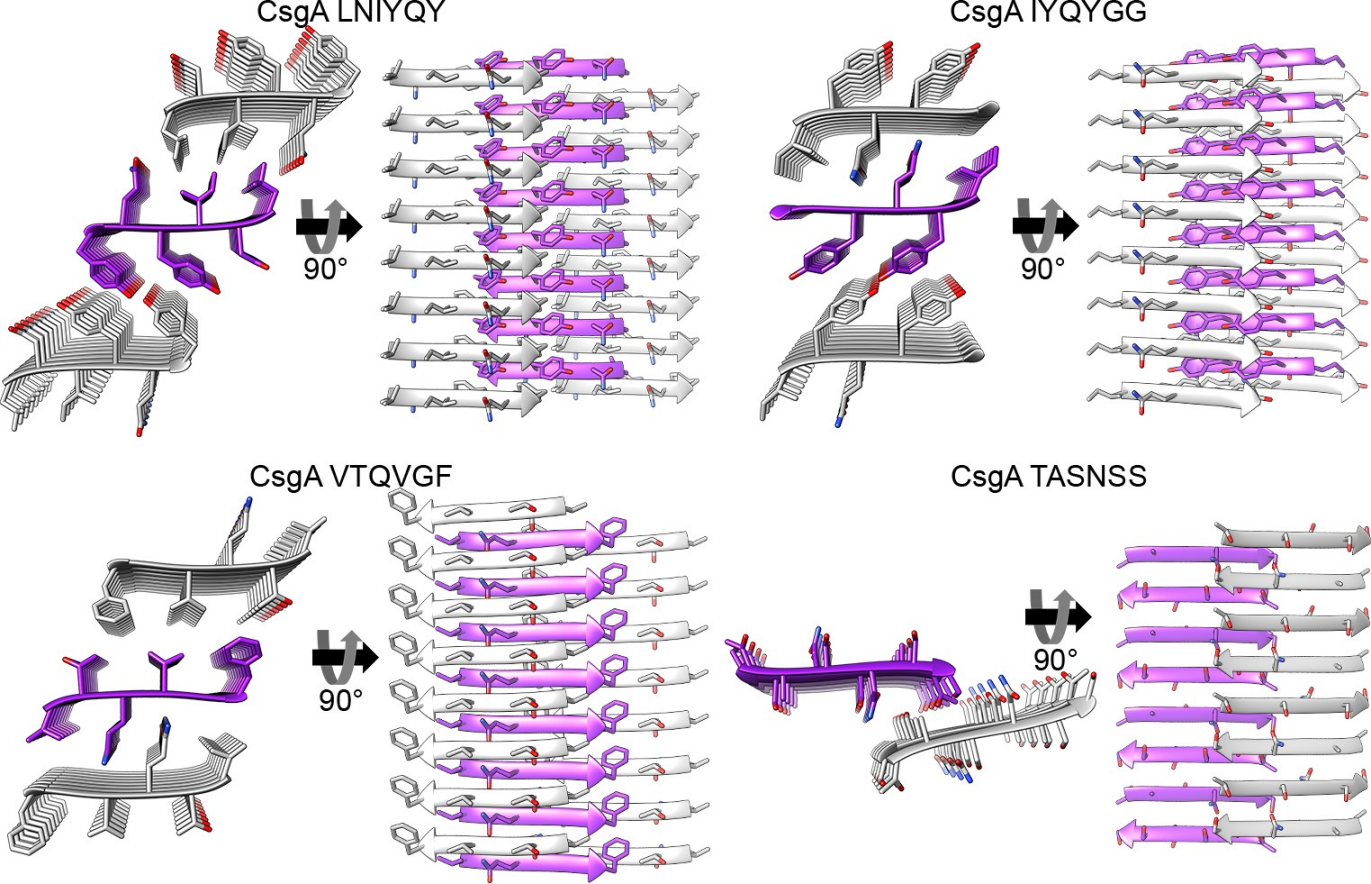
Crystal structures of CsgA spine segments. High-resolution crystal structures of CsgA segments are shown. In each structure, the left view is down the fibril axis, with residues shown as sticks, and the right view is perpendicular to the fibril axis, with β-strands, shown as ribbons, running horizontally. Eight layers of β-strands are shown, while actual fibrils contain thousands of layers. The carbons of each β-sheet are colored either gray or purple; heteroatoms are colored by atom type (nitrogen in blue, oxygen in red). The _45_LNIYQY_50_, _47_IYQYGG_52_ and _137_VTQVGF_142_ segments formed the classical class 1 steric zipper architecture of tightly mated parallel β-sheets with individual subunits (peptides) situated perpendicular to the fibril axis. Two possible dry interfaces in the crystal packing are displayed. The _129_TASNSS_134_ segment formed extended β-strands that stacked in an anti-parallel manner, but with a small contact area between two facing β-sheets and lacking a dry interface. S3–S6 Figs provide a detailed description of the four crystal structures.

To obtain atomic-resolution structural insight into curli fibrils, we solved the crystal structures of the four segments (Fig 1, S3–S6 Figs, S2 Table). _45_LNIYQY_50_ and _47_IYQYGG_52_, which are overlapping segments from the R1 repeat, adopted very similar structures. Both segments, as well as _137_VTQVGF_142_ (R5), adopted a classical amyloid steric zipper architecture, with two possible dry interfaces between paired β-sheets (Fig 1). The β-strands were oriented parallel to each other along the β-sheets. These three structures are class 1 steric zippers, as defined by Sawaya and Eisenberg according to the organization of the β-strands and β-sheets [24, 25]. In each of these dry interfaces, the chemical properties governing fibril stability, i.e., buried surface area and shape complementarity between sheets, resembled those of eukaryotic steric zipper structures (S3 Table). Correspondingly, the three segments formed fibrils that bound ThT (S1 and S2 Figs). Unlike the three spine segments that adopted tightly packed steric zipper architectures, _129_TASNSS_134_ adopted an anti-parallel β-sheet structure lacking a dry interface between mated sheets. The packing of the β-sheets most closely resembled a class 8 steric zipper [24], with a truncated interface between the two facing β-sheets.

### CsgA fibrils share structural characteristics with cross-β amyloids and seed the fibrillation of human Aβ

The structural features of full-length CsgA fibrils were analyzed using attenuated total-internal reflection Fourier transform infrared (ATR-FTIR) spectroscopy and showed a main peak at 1617 cm^-1^, corresponding to a rigid cross-β amyloid architecture [67–69] (S7 Fig). Furthermore, in accordance with a previous report [70], CsgA seeds accelerated the fibrillation of Alzheimer’s disease-associated Aβ_1–40,_ which have been shown to adopt canonical cross-β fibril architectures [20], resulting in massive fibril formation compared to Aβ_1–40_ alone (S8 Fig). The R1 segment _45_LNIYQY_50_ and the R5 segment _137_VTQVGF_142_ also induced fibrillation of Aβ_1–40_ (S8 Fig). The R1 segment _47_IYQYGG_52_ and the control segment _129_TASNSS_134_ showed a milder effect on the fibrillation rate of Aβ_1–40_ (S8 Fig).

### CsgA shares fibrillation inhibitors with Aβ

**Fig 2:**
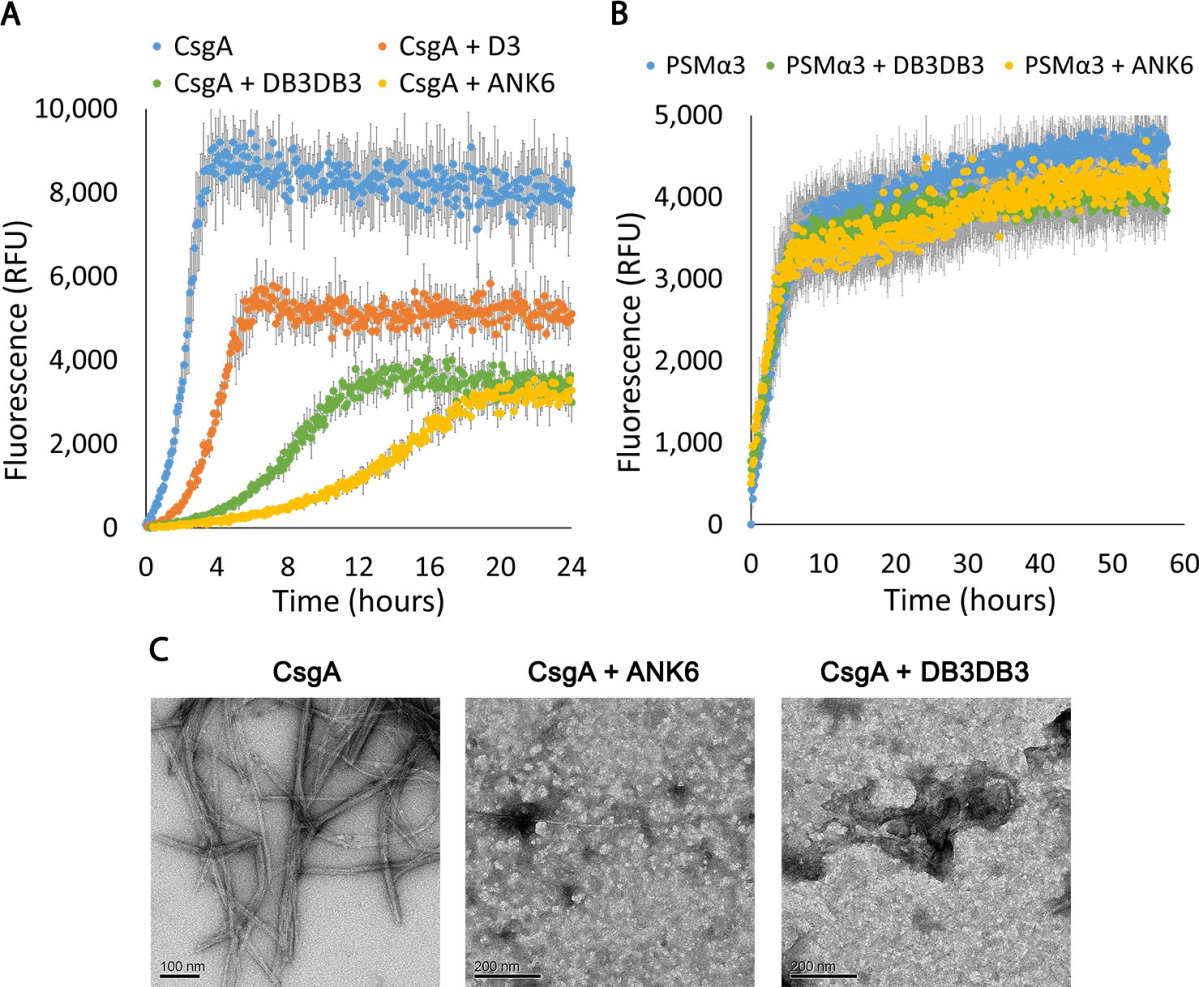
D-peptides inhibit fibrillation of CsgA but not of PSMα3. Mean fluorescence of ThT measurements in samples of CsgA (A) or PSMα3 (B), with or without the D-peptides, at a 1:5 molar ratio. Error bars represent standard error of the mean calculated from triplicates. ANK6 and DB3DB3 delayed fibril formation of CsgA and reduced the fluorescence signal, while demonstrating no effect on PSMα3 fibrillation. D3 showed a minor effect on CsgA (A). (C) Electron micrographs of CsgA incubated overnight, showing the formation of fibrils. In the presence of ANK6 or DB3DB3 (1:10 molar ratio of CsgA to D-peptides), only amorphous aggregates were observed. Scale bars are provided for each micrograph.

We next sought to investigate whether known inhibitors of pathological human amyloid formation can effectively inhibit CsgA fibrillation on the basis of this proposed structural similarity. We tested a group of synthetic D-enantiomeric peptides (referred to here as D-peptides) designed against Aβ [50–60]. Two of the D-peptides tested, ANK6 and DB3DB3 [55], were found to inhibit CsgA fibrillation in a dose-dependent manner. Specifically, freshly purified recombinant CsgA showed a characteristic ThT amyloid-fibrillation kinetics curve with a very short lag time, followed by rapid aggregation, while the addition of the D-peptides resulted in a lower fluorescence signal and a longer lag-time, indicative of delayed fibril formation (Fig 2A and S9 Fig). At 1:5 molar ratios of CsgA to inhibitor, ANK6 induced a longer lag time in CsgA fibrillation than DB3DB3, but both reaction mixes eventually reached similar maximal fluorescence intensities, which were significantly lower than that of CsgA without the D-peptides (Fig 2A). These data may reflect the ability of the D-peptides to induce different morphologies of the CsgA fibrils or interfere with one or more microscopic processes underlying the kinetics of CsgA fibrillation. Dose-dependent inhibition of CsgA fibrillation by D-peptides is shown in S9 Fig, where a significant inhibitory effect was already observed at 1:1 molar ratio of CsgA to the D-peptides. The TEM micrographs correspondingly showed mostly amorphous aggregates or co-precipitates of CsgA in the presence of ANK6 or DB3DB3 compared to fibrils of CsgA alone (Fig 2C). Both ANK6 and DB3DB3 were more potent fibrillation inhibitors than D3 (Fig 2A and S9 Fig), a prototype D-peptide previously shown to reduce amyloid deposits and inflammation and improve cognition in transgenic mouse models for Alzheimer’s disease [51]. In agreement with the hypothesis that the steric zipper segments serve as spines of CsgA fibrils, we found that the D-peptides also inhibited the fibrillation of the R1 _45_LNIYQY_50_ and R5 _137_VTQVGF_142_ spine segments in a dose-dependent manner (S10 Fig). Moreover, the same inhibitory series for the D-peptides (ANK6>DB3DB3>D3) was observed for the spines. Interestingly, these two segments showed the largest effect on enhancing fibrillation of Aβ_1–40_ (S8C Fig). In contrast to CsgA and its spine segments, ANK6 and DB3DB3 did not affect the fibrillation of the PSMα3 peptide secreted by the pathogenic *S*. *aureus* bacterium, which forms cross-α amyloid-like fibrils [27] (Fig 2B), suggesting that inhibition is dependent on the secondary structure of the fibril. Of note, TEM micrographs showed that these three D-peptides did not form fibrils (S1B Fig).

The effect of ANK6 and DB3DB3 on the secondary-structure transition of CsgA during fibrillation was assessed using time-dependent circular dichroism (CD) spectroscopy (S11 Fig). The CD spectra of freshly purified recombinant CsgA displayed a typical random coil configuration, with a detected minimum around 195 nm. After six hours of incubation, the spectra of CsgA showed a transition to a well-ordered β-sheet structure, with a distinctive maximum near 198 nm and minimum near 218 nm. The timescale of this transition was similar to that observed in a previous work [41]. The CD spectra of CsgA incubated with ANK6 (at 1:5 molar ratio) showed that CsgA retained a random coil configuration throughout the 18 hours of incubation (S11 Fig), indicating inhibition of CsgA fibrillation.

**Fig 3:**
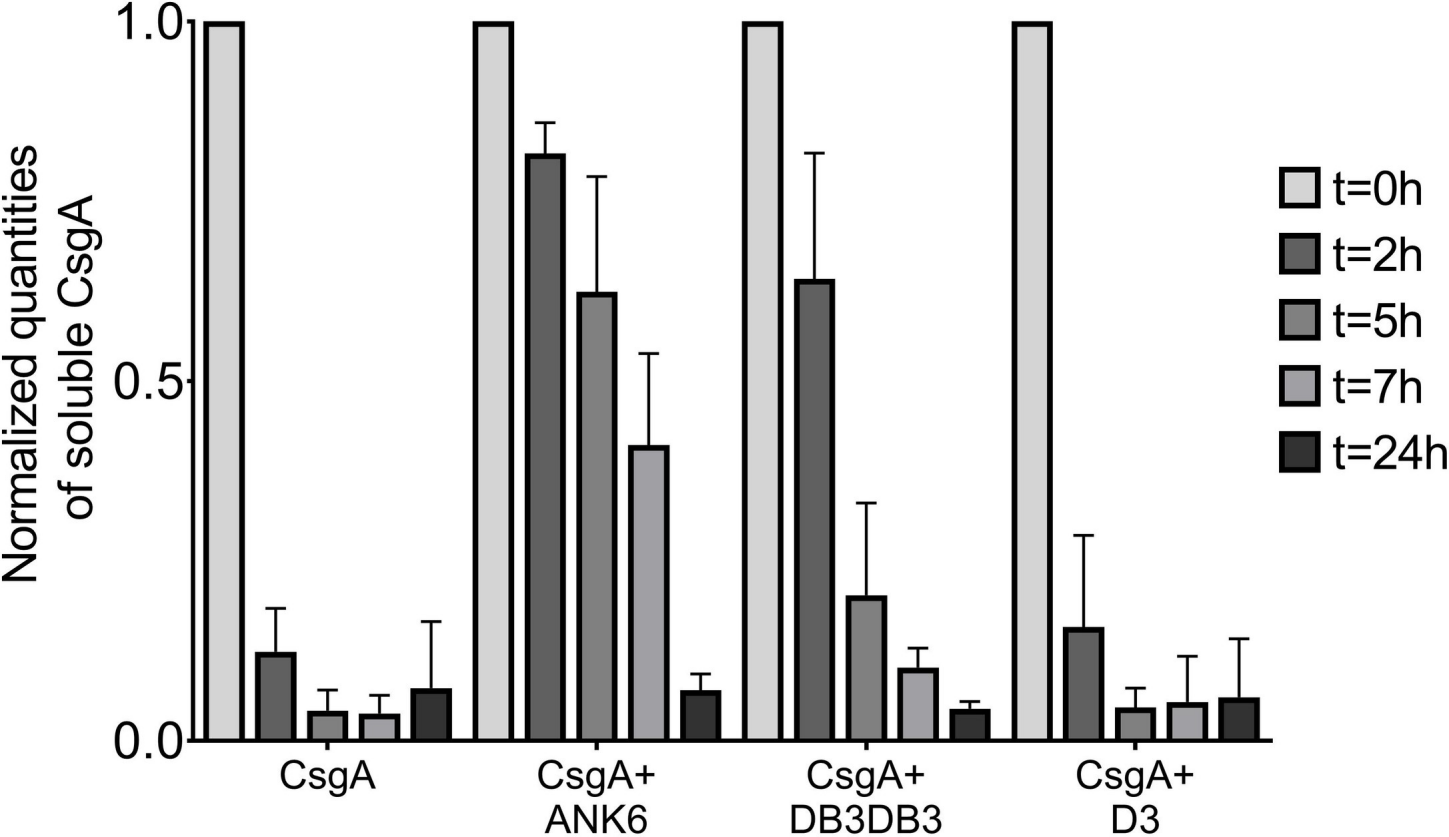
The D-peptides stabilize soluble CsgA and inhibit fibrillation. The effect of D-peptide inhibitors on CsgA fibrillation at 5:1 molar ratios, as quantified based on CsgA ability to migrate on a 15% SDS-PAGE (exemplified in S12 Fig). CsgA incubated with ANK6 and DB3DB3 showed a prolonged soluble state, suggesting inhibition of the rapid formation of SDS-insoluble fibrils. The graph represents three experimental repeats. The experiments were repeated at least three times on different days, all giving similar results.

CsgA fibrils are resistant to sodium dodecyl sulfate (SDS) solubilization [41, 71]. We therefore examined the effect of the D-peptide inhibitors on CsgA fibrillation by assessing its migration on a polyacrylamide gel (Fig 3 and S12 Fig). We first assessed the oligomeric state of freshly purified recombinant CsgA using size exclusion chromatography coupled with multi-angle light scattering (SEC-MALS) (S13 Fig). This analysis showed that the majority of freshly purified CsgA was in the monomeric state. A minor population existed as hexameric oligomers. The presence of some oligomers at these early time points was not surprising, considering the rapid fibrillation kinetics observed for CsgA, but it was still unclear whether these hexamers play a role in CsgA fibrillation. Interestingly, the formation of pentamers or hexamers as the smallest populated assembly species, has been described for Aβ as well [72]. As expected, freshly purified soluble CsgA, but not incubated CsgA, migrated in SDS polyacrylamide gel electrophoresis (SDS-PAGE) (Fig 3 and S12 Fig). In the presence of DB3DB3, and to a greater extent ANK6, the soluble CsgA state was stabilized, suggesting a robust effect of the D-peptide inhibitors on CsgA fibrillation in accordance with the ThT, TEM (Fig 2 and S9 Fig) and CD results (S11 Fig).

### D-peptides reduce *Salmonella typhimurium* biofilm formation

**Fig 4:**
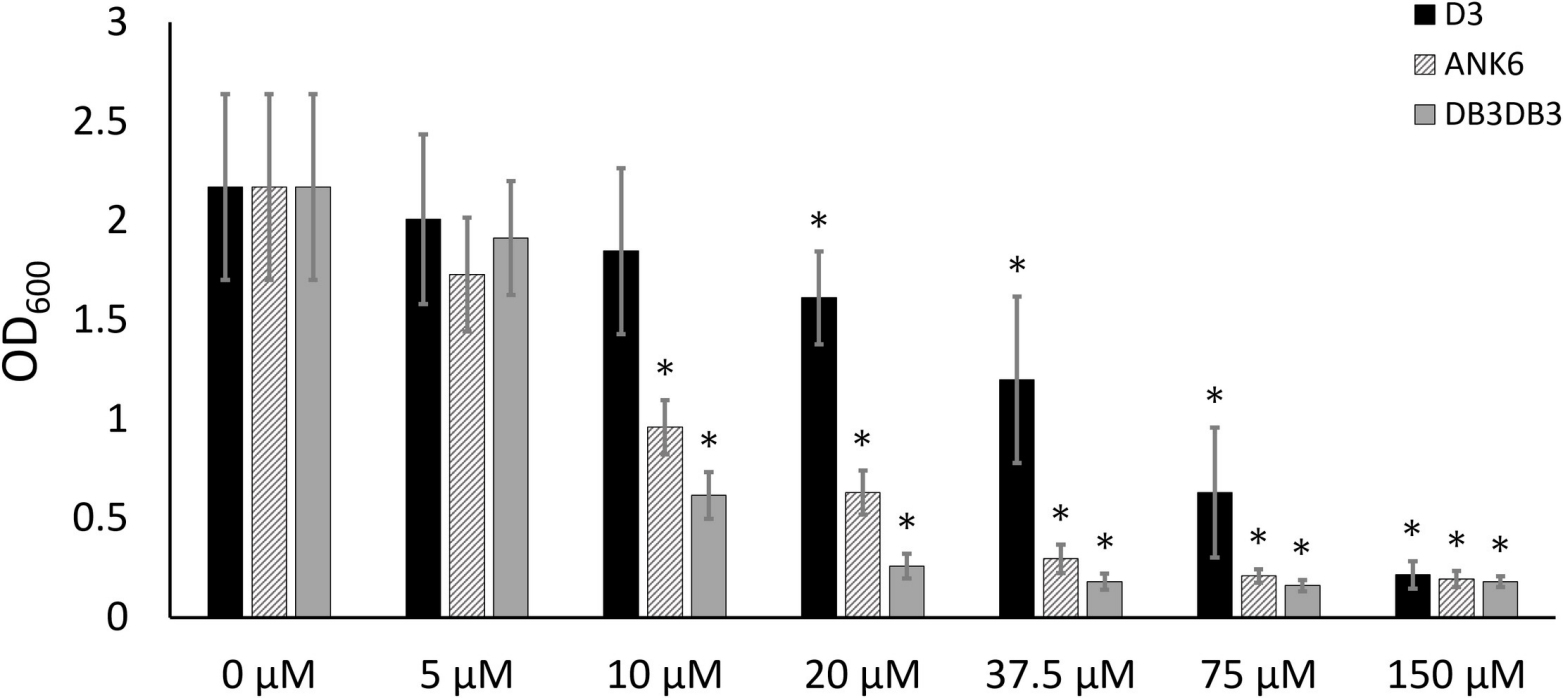
D-peptides reduce formation of *S*. *typhimurium* static biofilm. **D** Static *S*. *typhimurium* MAE52 biofilm production assessed after a 48-hour incubation at 30°C, in the presence or absence of increasing concentrations of D-peptides, quantified using crystal-violet staining measured by optical density at 600 nm (OD_600_). Statistical significance was analyzed using the Mann-Whitney non-parametric test. Error bars represent standard deviations. * p<0.05 compared to control (no inhibitor added).

**Fig 5:**
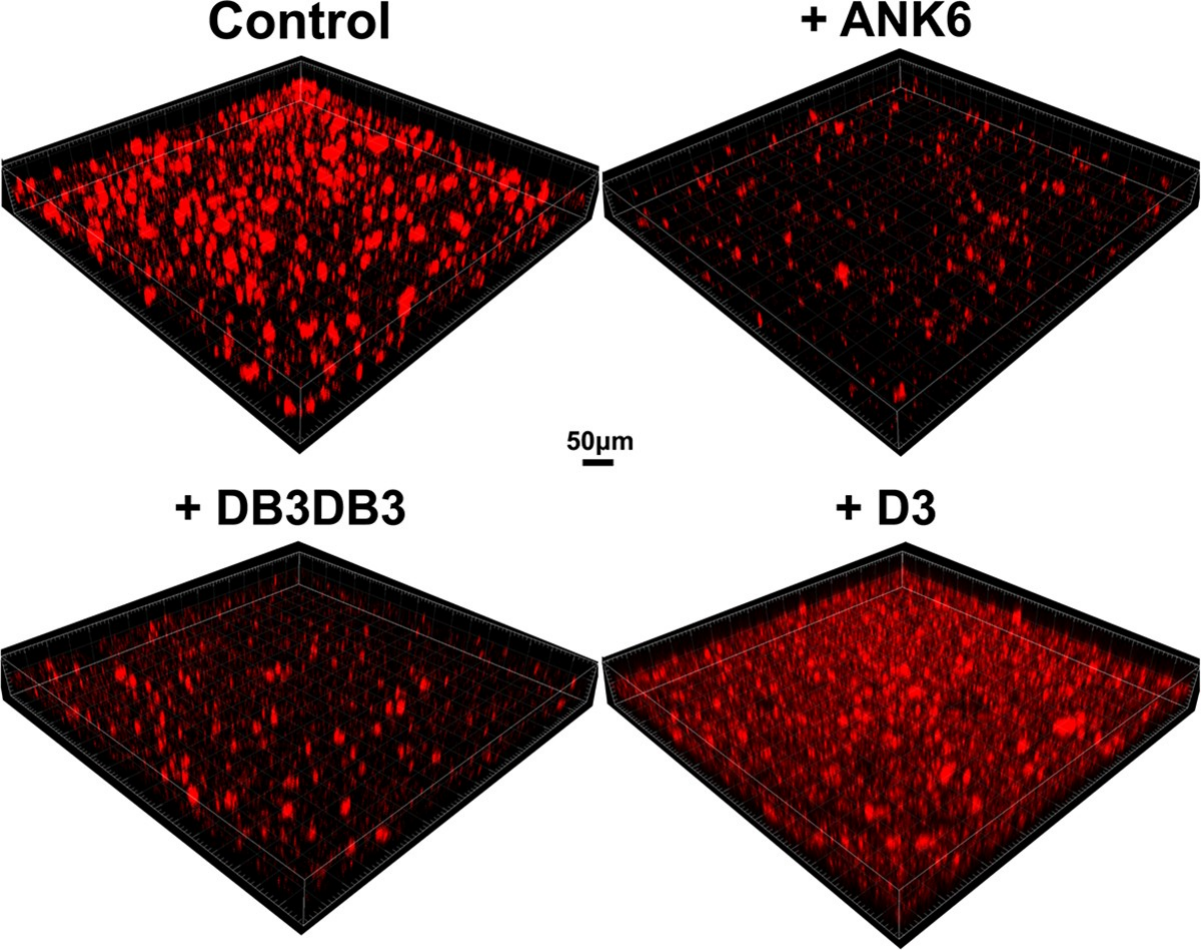
D-peptides reduce formation of surface-attached *S*. *typhimurium* static biofilm. Confocal microscopy images showing surface-attached static *S*. *typhimurium* MAE52 biofilms formed after 48 hours at 30°C, in the absence (control) or presence of 10 μM D-peptides. Samples were stained with propidium iodide.

Treatment with ANK6 and DB3DB3 reduced the total static biofilm biomass formation of *S*. *typhimurium* MAE52 (a constitutive curli fimbria and cellulose producing strain) in a dose-dependent manner, as shown by lower crystal violet staining of the biofilm (Fig 4). Both DB3DB3 and ANK6 showed a significant inhibitory effect at a dose of 10 μM, with DB3DB3 producing a more pronounced effect. The D3 peptide only slightly affected biofilm formation at a concentration of 20 μM and was less effective than the other two D-peptides, in accordance with its in-vitro inhibition of CsgA fibrillation. No effect on bacterial growth was observed in the presence of up to 150 μM of any of the three peptides, demonstrating that the observed impact on biofilm mass was unrelated to a bacteriostatic or bactericidal effect (S14 Fig). Confocal microscopy images showing biofilm cells stained with propidium iodide confirmed the significant reduction in the formation of an otherwise abundant surface-attached biofilm of *S*. *typhimurium* by the addition of 10 μM DB3DB3 or ANK6 (Fig 5). D3 (10 μM) had no significant effect on the biofilm, in agreement with the crystal violet assay.

### D-peptide effects on biofilm formation are amyloid-related

**Fig 6:**
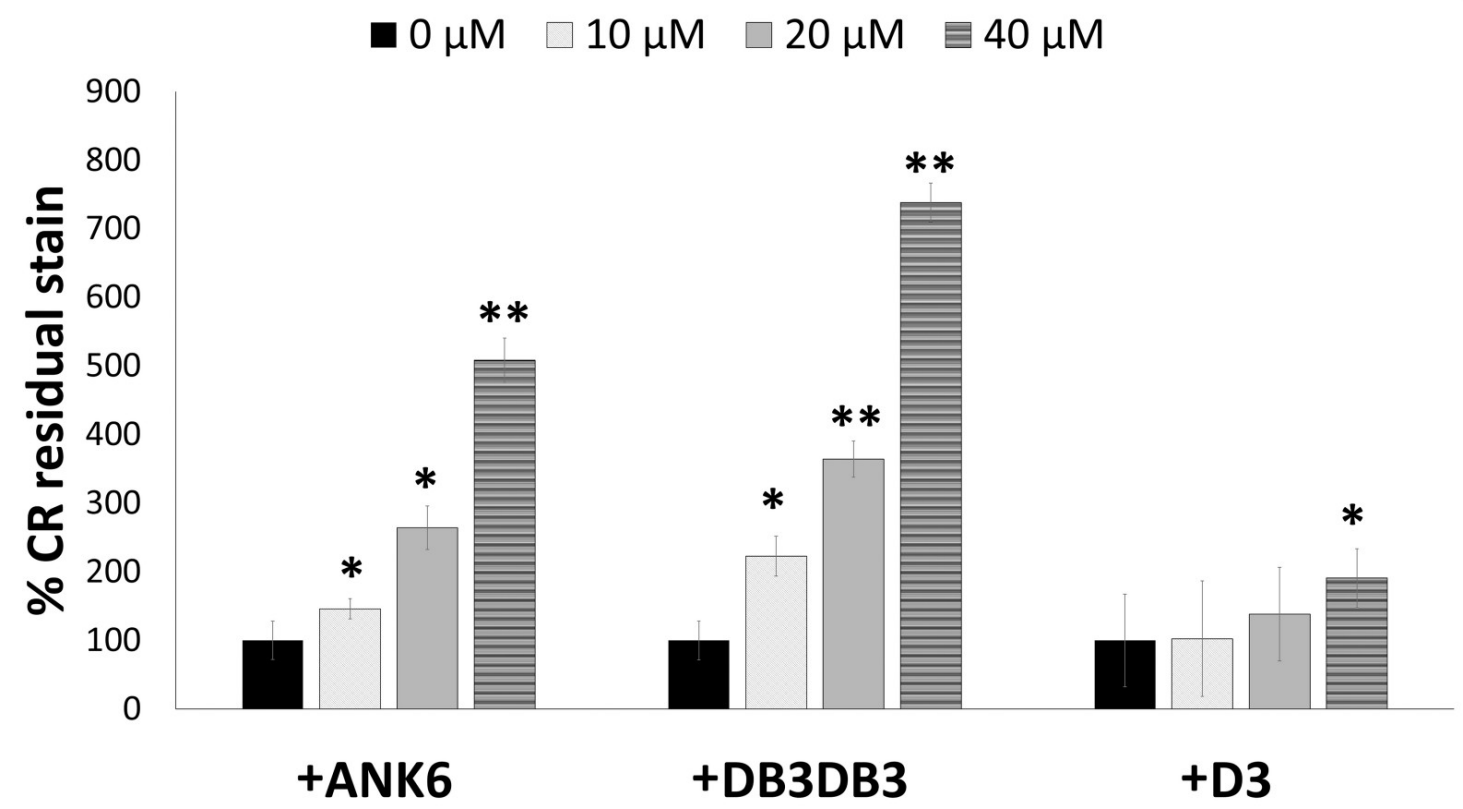
D-peptides reduce curli production in *S*. *typhimurium* biofilm. Quantification of the effect of D-peptides on curli fibril formation in the bacteria. Shown are the levels of measured residual CR in the agar following the removal of the biofilm colony of the *S*. *typhimurium* MAE150 cellulose-deficient mutant. Control colonies that were not exposed to the D-peptides were assigned 100% CR residual stain remaining in the agar. A higher percentage of residual CR stain indicates less CR adsorption by the colony and hence, reduced curli fibril formation. Error bars represent standard deviations. * p<0.05 and ** p<0.005 compared to control (no inhibitor added). CR plates from one of the experiments are shown in S15B Fig.

In order to confirm that the effect of the D-peptides on biofilm biomass is related to inhibition of amyloid formation, *S*. *typhimurium* MAE52 cells were grown on agar supplemented with congo red (CR), a dye which is known to stain amyloids, including curliated whole cells [8]. The resulting biofilm was reddish, indicating adsorption of the dye from the agar (S15A Fig). Pre-addition of ANK6 and DB3DB3 to the bacteria showed a dose-dependent discoloration at the center of the biofilm colony (where the suspension was initially placed on the agar), indicating less CR adsorption (S15A Fig) and hence less fibril formation. Since CR also stains secreted cellulose in biofilm [73], the *S*. *typhimurium* MAE150 mutant (MAE52 derived strain that does not express cellulose) was grown on agar under the same conditions, to verify the effect of the D-peptides on curli fibril formation. To comparatively quantify *S*. *typhimurium* whole-cell curliation in the presence and absence of the D-peptides, the colonies were removed from the CR-supplemented agar and the concentration of CR that was not adsorbed by the curli-producing bacterial cells but remained in the agar, was measured (Fig 6 and S15B Fig). DB3DB3 was most effective in reducing curli fibril formation, followed by ANK6, while D3 only elicited an effect at a dose of 40 μM. These results are in agreement with the effect of the D-peptides on static biofilm formation (Fig 4). Overall, ANK6 and DB3DB3 showed inhibitory effects on CsgA fibrillation in-vitro, and on curli fibrillation and biofilm formation in the bacteria.

## Discussion

### Cross-β steric zipper fibrils likely stabilize and structure biofilms

The curli biogenesis machinery is designed to secrete, nucleate and elongate extracellular amyloid fibrils that participate in biofilm formation and stability [3–9]. While the amyloidogenic properties of the major curli subunit, CsgA, have been investigated in detail, high-resolution structural information on CsgA fibrils is lacking. In this study, we elucidated atomistic structural features of CsgA spine segments. Specifically, the R1 _45_LNIYQY_50_ and _47_IYQYGG_52_ segments and the R5 _137_VTQVGF_142_ segment formed fibrils that bound the amyloid indicator dye ThT and formed canonical amyloid class 1 steric zippers (Fig 1 and S1–S5 Figs). These segments contain Gln49 or Gln139 (marked in bold in _45_LNIY**Q**Y_50_, _47_IY**Q**YGG_52_, and _137_VT**Q**VGF_142_), which are critical for fibrillation and cannot be mutated to asparagine without interfering with curli assembly [34]. In contrast, _129_TASNSS_134_ does not contain any residues that have been shown to act as sequence determinants of CsgA fibrillation [34], and moreover, Ser133 is nonessential for fibrillation [34]. Furthermore, in agreement with its atypical crystal structure, _129_TASNSS_134_ formed scarce and more amorphous structures (S1A Fig), and did not bind ThT (S2 Fig). These observations demonstrated that not all segments that are predicted to have high aggregation propensities actually form steric zippers. Here, only those segments which were considered likely to play a role in nucleating fibrillation according to biochemical evidence, formed steric zippers. We postulate that the three steric-zipper forming spine segments from R1 and R5 contribute to scaffolding and stabilizing the robust curli amyloid architecture.

The CsgA steric zipper spine structures closely resemble those of pathological human amyloids (S3 Table). Extensive structural studies on pathological amyloids have suggested that their cross-β signatures result from steric zipper-like structures in the fibril spine formed from one or more short segments of the protein [74–76]. We therefore hypothesize that the CsgA steric zipper spine segments contribute to the structured core of the CsgA fibril and allow for the formation of cross-β fibrils by full-length CsgA [2, 37, 39, 41]. Cross-β ultra-stable structures have been mechanically characterized to be as stiff as silk and as strong as steel [77]. In bacteria, these fibrils likely stabilize and structure biofilms, thereby rendering the bacterial communities more resilient and resistant to antibiotics [2, 6, 9, 10]. Similarly, steric zipper structures of spine segments were suggested to form the cores of the cross-β fibrils of PSMα1 and PSMα4 found in the biofilm of *S*. *aureus* [30]. Together, our findings suggest that segments capable of forming steric zippers may be a structural hallmark of biofilm-associated microbial amyloids as well as their disease-associated counterparts, supporting a structural building block that is conserved from bacteria to human.

### Structural models of full-length CsgA fibrils

A previously proposed structural model of full-length CsgA suggested that the fibrils adopt a β-helix or β-solenoid-like fold. This was based on a model suggested for *Salmonella* CsgA [35] and on information obtained from solid-state NMR and electron microscopy [39, 42] that were more consistent with a β-helix-like structure rather than with in-register parallel β-sheets, although the authors note that the data were insufficient to definitively confirm the adoption of such a structure [39]. This arrangement was also supported by a computational model suggesting that in such a β-helical fibril, each turn corresponds to one repeat sequence of CsgA, forming two β-strands connected by a loop, which ultimately creates a “rectangular” hydrophobic core [78]. The elongation of the structure is achieved by intermolecular stacking along the fibril axis mediated via the R1 and R5 repeats [78]. We herein suggest an alternative model in which fibril formation is nucleated and stabilized via several spine segments in the R1 and R5 repeats. In this model, only specific regions form β-sheets and structure the fibril in contrast to the entire protein being structured as in the β-helix model. The lack of CsgA fibril polymorphism [33] supports both the β-helix model and the spine-based model. In the β-helix model, polymorphism may be averted as the entire protein is involved in structuring the fibril. In the spine-based model, polymorphism may be avoided due to the sequence specificity of nucleation. The gatekeeping residues in the R2-R4 repeats [36] might prevent segments within these repeats from serving as spines, thereby allowing for specific nucleation sites to mediate homogenous fibril assembly.

ATR-FTIR spectroscopy was used to obtain more insight on the structural properties of full-length CsgA fibrils. Previous analyses of amyloids [67, 79–82] revealed a signature FTIR spectral peak between 1611 and 1630 cm^−1^, while native β-sheet-rich proteins [67] showed a peak at higher wavelengths of 1630–1643 cm^−1^. Often, a shift from higher to lower wavelengths is observed while monitoring fibril formation [80], which indicates the assembly of longer and planar sheets [67, 83], an increase in the number of β-strands in the β-sheet and/or the formation of stronger hydrogen bonding, typical of extremely stable amyloid fibrils [68, 80]. In contrast to canonical amyloids, mature fibrils of the yeast prion HET-s(218–289) fragment, which was shown by solid-state NMR to form a β-solenoid-like fold [84], showed an FTIR peak at 1630–1631 cm^-1^ [85]. This peak wavelength is at the threshold between those defining amyloids and native β-sheet proteins [67]. CsgA fibrils showed a peak at 1617 cm^-1^ (S7 Fig), which aligns with the range of peaks corresponding to rigid cross-β amyloid fibrils [67–69]. A main FTIR peak at 1617 cm^-1^ was also shown for amyloid fibrils of apomyoglobin [86] and of γD-crystallin [87]. A previous work showed a main FTIR peak for CsgA at 1623 cm^-1^, also within the lower wavelength range [41]. Overall, the FTIR spectra of CsgA fibrils support the formation of stable cross-β fibrils of in-register, tightly mated sheets. Moreover, we expect that a β-helix fold, which encompasses the entire CsgA sequence, would originate from a folded or partially folded monomers, yet, freshly purified recombinant CsgA is disordered in nature [41] (S11 Fig). Nevertheless, high-resolution structural work on full-length CsgA is needed to accurately describe the architecture of the fibrils, considering the possible existence of novel types of fibril organizations.

### D-enantiomeric peptides designed for Alzheimer’s disease prevented biofilm development

The D-enantiomeric peptide D3 was identified from a mirror image phage display selection against monomeric Aβ_42_ with the intention to stabilize monomeric Aβ_42_ and to shift equilibria between various Aβ assembly species away from toxic Aβ oligomers. In vivo, treatment with D3 led to reduction of amyloid plaque load and, more importantly, to improvement of cognition in transgenic AD mice. In vitro, D3 converts toxic Aβ oligomers into non-toxic amorphous co-precipitates of D3 and Aβ [53, 54, 88]. DB3DB3 and ANK6 are D3 derivatives designed to stabilize Aβ monomers more efficiently than D3. They were indeed shown to bind Aβ monomers with increased affinity and to eliminate oligomers more efficiently compared to D3 [55, 58]. Inspired by the structural resemblance of spine segments of CsgA to those of human pathological amyloids, we tested the D-enantiomeric peptides [50–55], and found ANK6 and DB3DB3 to inhibit fibrillation of full-length CsgA, slowing its transition from an unstructured soluble configuration to insoluble fibrils (Figs 2 and 3 and S9, S11 & S12 Figs). Since CsgA fibrillation is extremely fast, we assume that the D-peptides bind and stabilize CsgA monomers, and possibly forming amorphous co-aggregates (Fig 2C) that are SDS-soluble (Fig 3). These D-peptides also inhibit the fibrillation of the CsgA spine segments from the R1 and R5 CsgA repeats (S10 Fig), suggesting that these regions can serve as binding sites within the full-length CsgA. As these segments contain positions important for fibrillation and have high aggregation propensity, binding of the D-peptides to these regions could occlude nucleation and delay fibrillation. We thereby suggest that the steric-zipper spine segments serve as critical determinants of fibrillation. The ability of the D-peptides to inhibit both CsgA and Aβ provides further support for the claimed structural similarity between their targets. Moreover, ANK6 and DB3DB3 did not affect the fibrillation of the cytotoxic *S*. *aureus* PSMα3, which forms cross-α amyloid-like fibrils [27], demonstrating specificity in structural properties targeted by the inhibitors.

ANK6 and DB3DB3 significantly reduced static biofilm formation of *Salmonella typhimurium* in a curli-dependent manner (Figs 4–6 and S15 Fig), in agreement with their effects on CsgA fibrillation in-vitro. Similarly, other small molecules and peptidomimetics that have been shown to interfere with the in-vitro assembly of amyloids secreted by *E*. *coli*, *B*. *subtilis* and other bacteria, prevent biofilm formation and pilus biogenesis [6, 71, 89–95]. Moreover, curlicides (compounds acting against the curli amyloid) attenuated uropathogenic *E*. *coli* in a murine model of a urinary tract infection [90]. Furthermore, two such identified compounds also inhibited conversion of the yeast New1 protein to the prion state [95]. Here, we offer a novel strategy for disrupting curli amyloid formation, and prompt further investigation into the promiscuity of a class of anti-amyloid therapeutics as antivirulence agents targeting amyloid-structured biofilms.

### Can CsgA influence neurodegenerative diseases?

Amyloid protein folding and aggregation patterns are highly conserved through evolution and appear in all kingdoms of life [61]. Amyloids have even been suggested to serve as prebiotic replications of information-coding molecules [96]. The best-known pathological manifestation of amyloid self-assembly is in association with neurodegenerative diseases, which involve the formation of transmissible, self-propagating prion-like proteins [97, 98]. Molecular structures shared between amyloids of different species may be involved in the creation of these prion-like agents through molecular mimicry [61], raising concerns regarding the exposure of humans to various food sources and microbes that contain amyloids [99–101]. Indeed, microbial amyloids interact with the amyloids of host systems [6, 102], putatively providing some immune-evasive and survival strategies [11, 103], and have been suggested to contribute to the pathology of aggregation diseases [102, 104–113]. This phenomenon may underlie the ability of seeds of amyloid fibrils from one species to nucleate monomers from another species [70, 114–116]. For instance, infecting the brains of transgenic mice with *Salmonella Typhimurium* elicited rapid Aβ deposition closely co-localized with the invading bacteria [117]. Exposure to curli-producing *E*. *coli* enhanced α-synuclein aggregation involved in Parkinson’s disease in aged rats and Caenorhabditis elegans [116]. Silk from *Bombyx mori*, Sup35 from *Saccharomyces cerevisiae*, and curli from *E*. *coli* were shown to promote secondary amyloidosis disease in mice [115]. Murine amyloidosis was also accelerated by dietary ingestion of both purified amyloid fibrils and tissue homogenates that contain amyloid fibrils [99]. Several microorganisms, including herpes simplex virus type 1 and various periodontal pathogens, were associated with dementia and brain lesions in Alzheimer’s disease and islet lesions in type 2 diabetes [109, 111, 118–123]. These and other reports on the connection between microbes and amyloid diseases call for comprehensive molecular-level analyses of the interactions between microbial and human amyloids. Here, we showed that CsgA and spine segments from R1 and R5 can cross-seed fibrillation of Alzheimer’s disease-associated Aβ (S8 Fig), in accordance with a previous report [70].

Epitaxial heteronucleation necessitates some degree of structural similarity between participating amyloids, suggesting that full-length CsgA adopts a fibril architecture sufficiently similar to that of Aβ to effectively template fibril elongation [70, 124]. Nucleation is expected to be specific, as for example, islet amyloid polypeptide protein fibrils were unable to seed CsgA [125]. The influence of curli-producing bacteria on human neurodegenerative diseases could originate from the nucleation, acceleration or deterred fibrillation of human amyloids. Alternatively, the bacteria may affect the immune system, leading to inflammation and stress that are correlated with neurodegenerative diseases [126, 127]. Future work is needed to characterize additional microbial amyloids at the molecular and atomic levels, study their inter-species interactions and modulate their activities [128].

## Materials and methods

### Peptides and reagents

Curli peptide segments LNIYQY, IYQYGG, VTQVGF, and TASNSS from CsgA (UniProt accession number P28307), and TAIVVQ from CsgB (UniProt accession number P0ABK7) were used. The peptides were synthesized with unmodified termini for crystallography, or with fully or semi-capped termini (acetylated in the N-terminus and amidated in the C-terminus), as specified, for the other assays. The curli segments, PSMα3 (UniProt accession number P0C805), Aβ_1–40_ (all custom synthesis) at >98% purity were purchased from GL Biochem (Shanghai) Ltd. The tested D-peptide fibrillation inhibitors consisted of D-enantiomeric amino acids, and were C-terminally amidated. The sequences were as follows: ANK6: RKRIRLVTKKKR-NH2, DB3DB3: RPITRLRTHQNRRPITRLRTHQNR-NH2 and D3: RPRTRLHTHRNR-NH2 (S1 Table). The D-peptides (custom synthesis) at >95% purity were purchased from either GL Biochem (Shanghai) Ltd., peptides&elephants (Potsdam, Germany), or JPT Peptide Technologies (Berlin, Germany). Thioflavin T (ThT), congo red and crystal violet were purchased from Sigma-Aldrich. Dimethyl-sulfoxide (DMSO) was purchased from Merck. Ultra-pure water was purchased from Biological Industries.

### Preparation of D-peptides

D-peptides inhibitors (ANK6, DB3DB3 and D3) were solubilized in ultra-pure water and their concentrations were calculated using a spectrophotometer Nanodrop 2000c instrument (Thermo), at an absorbance of 205 nm, with the specific extinction coefficient calculated for each peptide by the ‘protein parameter calculator’ [129] (http://nickanthis.com/tools/a205.html).

### Computational prediction of amyloid spine segments

Amyloidogenic propensities of CsgA and CsgB segments were predicted using combined information from several computational methods, including ZipperDB [62], Tango [63, 64], Waltz [65] and Zyggregator [66].

### CsgA expression and purification

The protocol for CsgA expression and purification was adapted from Wang et al. [36]. A plasmid containing the CsgA sequence cloned into pET11d with a C-terminal His_6_-tag, was kindly provided by the Chapman lab (University of Michigan, USA) [36]. The plasmid was transformed to *E*. *coli* BL-21 cells, which were then grown overnight in 25 ml Luria-Bertani (LB) medium supplemented with 50 μg/ml ampicillin, and further diluted into 700 mL of the same medium, and then incubated at 37°C with 220 rpm shaking, until OD_600_ was 0.8–0.9. CsgA expression was induced with 0.5 mM isopropyl β-D-1-thiogalactopyranoside (IPTG) for 1 h. Bacterial cell pellets were harvested by centrifugation at 4,500 rpm for 25 min and stored at -20°C. Thawed cell pellets were resuspended in 25 ml lysis buffer (8 M guanidinium HCl, 50 mM potassium phosphate buffer pH 7.3) and incubated at room temperature (RT), with agitation, for 18–24 h. The supernatant was separated by centrifugation at 10,000 g for 25 min and incubated with 1.6 ml HisPur cobalt resin beads (Thermo scientific) equilibrated with lysis buffer, at RT with agitation, for 1 h. The mixture was loaded on a disposable polypropylene column at 4°C and washed with 10 ml of 50 mM potassium phosphate buffer pH 7.3, followed by another column wash with the same buffer supplemented with 12.5 mM imidazole. CsgA was eluted with 125 mM imidazole in 50 mM potassium phosphate buffer pH 7.3. Freshly purified CsgA was filtered using a 30 kDa cut-off column at 4°C (Amicon Ultra-4, Sigma-Aldrich) to remove insoluble protein aggregates and seeds. Imidazole was removed by desalting the protein solution at 4°C using a Zeba spin 7K desalting column (ThermoFisher Scientific), into 50 mM potassium phosphate buffer pH 7.3. CsgA concentration was determined by measuring absorption at 280 nm, calculated with a molar extinction coefficient of 11,460 M^-1^ cm^-1^, as determined via the Expasy server (http://web.expasy.org/cgi-bin/protparam/protparam). The identity of CsgA was confirmed by Western blot, using anti-6X His tag antibody.

### Multi-angle light scattering size exclusion chromatography analysis to determine oligomerization state

Multi-angle light scattering size exclusion chromatography (SEC-MALS) analysis of freshly purified CsgA was performed to determine the accurate molecular weight and the oligomerization state of soluble CsgA. CsgA was concentrated to 1.8 mg/ml, using a 3 kDa cut-off spin column at 4ºC (Amicon Ultra-4, Sigma-Aldrich). SEC was performed over a size-exclusion column (Superdex 75 10/300) operated by AKTA avant. MALS was performed with miniDAWN TREOS (WYATT Technology) and its companion Optilab T-rEX (WYATT Technology) dRI detector. Characterization and analysis of the SEC-MALS results were performed using ASTRA software (WYATT Technology).

### Fibrillation assays

#### Transmission electron microscopy (TEM) to visualize CsgA fibrils

TEM was performed to visualize CsgA fibrils, and to test the effect of the D-peptide inhibitors. CsgA was purified as described above, and 30 μM was incubated overnight, at 25°C, with 300 rpm shaking in a plate reader, with and without 300 μM D-peptides (1:10 molar ratio). Five-microliter samples were then applied directly onto copper TEM grids with support films of Formvar/carbon (Ted Pella), which were charged by glow-discharge (PELCO easiGlow, Ted Pella) immediately before use. Grids were allowed to adhere for 2 min and negatively stained with 5 μl 2% uranyl acetate solution. Micrographs were recorded using a FEI Tecnai G2 T20 S-Twin transmission electron microscope at an accelerating voltage of 200 KeV, or using a FEI Tecnai T12 G2 transmission electron microscope operated at an accelerating voltage of 120 kV.

#### Thioflavin T kinetic assays of CsgA

Thioflavin T (ThT) is an extensively used “gold standard” stain for identifying and exploring formation kinetics of amyloid fibrils, both in-vivo and in-vitro. Fibrillation curves in the presence of ThT commonly show a lag time for the nucleation step, followed by rapid aggregation. Fibrillation kinetics of CsgA, in the presence or absence of D-peptide inhibitors, was monitored using this approach. Freshly purified CsgA in 50 mM potassium phosphate buffer, pH 7.3, was mixed with filtered ThT from stock made in ultra-pure water diluted in the same buffer, and with D-peptides from stock made in ultra-pure water. Final concentrations in the reaction wells were 15 μM CsgA mixed with 0, 15, 75 or 150 μM D-peptides, and 20 μM ThT in a final volume of 100 μl. The control wells included all the components except for the D-peptides, which were replaced by an equivalent volume of pure water. The reaction mixture was carried out in a black 96-well flat-bottom plate (Greiner bio-one) covered with a thermal seal film (EXCEL scientific) and incubated in a plate reader (CLARIOstar, BMG Labtech), at 25°C, with orbital shaking at 300 rpm, for 30 sec before each measurement. ThT fluorescence was recorded every two minutes using an excitation of 438±20 nm and an emission of 490±20 nm. The measurements were conducted in triplicates, and the entire experiment was repeated at least three times.

#### Thioflavin T kinetic assay of Aβ_1–40_ fibrillation seeded by CsgA and its segments

Fibrillation of Aβ_1–40_ was assessed by monitoring ThT kinetics, in the presence or absence of fibril seeds of CsgA or its segments. CsgA fibrils were obtained by incubation of freshly purified CsgA at a concentration of 10 μM for one week at 37°C with shaking at 300 rpm. The fibrils were washed three times in ultra-pure water by centrifugation at 17,000xg for 10 minutes and gently discarding the supernatant. The fibrils were then re-suspended in 50mM potassium phosphate buffer pH 7.3. CsgA spine segment (Ac-VTQVGF-NH_2_, Ac-LNIYQY-NH_2_, Ac-IYQYGG-NH_2_ and Ac-TASNSS-NH_2_) fibrils were obtained from 5mM solutions of 50% DMSO in ultra-pure water incubated for 2–3 days at 37°C with shaking at 300 rpm. Following incubation, the fibrils of CsgA and its segments were each sonicated on ice by a VC750 VibraCell tip sonicator (sonics, CT, USA) set at 30% amplitude with three 15 sec bursts at 50 sec intervals.

Preceding fibrillation assays, Aβ_1–40_ was pretreated in order to dissolve potential aggregates in the lyophilized powder. It was dissolved to 1 mM in 1,1,1,3,3,3- hexafluoro-2-propanol (HFIP), bath sonicated at RT for 10 min, incubated for 1 h at 37°C, dried using a SpeedVac (RVC 2–18 CDplus, CHRIST) and stored at *-*20°C prior to use. Pretreated Aβ_1–40_ aliquot was re-dissolved to 1mM in ultra-pure water supplemented with 10 μL of HCl 1M to reach full solubility. The solution was bath sonicated at RT for 10 min, and diluted to 50 μM in 50 mM potassium phosphate buffer pH 7.3. The ThT kinetic assay was carried out as described above with 50 μM pretreated Aβ_1–40_, 200 μM ThT and with or without the CsgA or spine segments fibril seeds, each added separately to a final concentration of 0.2, 1 or 5% vol. of the entire reaction. The measurements were performed in triplicates, and the entire experiment was repeated at least three times. At the end of the kinetics assay, samples were taken and TEM grids were prepared and visualized as described above.

#### Thioflavin T kinetic and TEM assay of CsgA spine segments

CsgA segments with capped termini (acetylated in the N-terminus and amidated in the C-terminus) were used to mimic their chemical nature in the full-length protein. For TEM visualization, the peptides were dissolved to 1 mM in DMSO and incubated at 37ºC, with 300 rpm shaking, for several days. TEM grids were prepared and visualized as described above. For the ThT assay, the peptides were dissolved in DMSO to 10 mM, and stored at -20ºC until use. The peptides were thawed and diluted directly into a reaction buffer comprised of 50 mM potassium phosphate buffer, pH 7.3, mixed with filtered ThT from stock made in ultra-pure water. The final concentrations were 100, 150, 200 or 500 μM of CsgA segments and 20 μM ThT in a final volume of 100 μL per well. The ThT reaction was conducted as described above. The measurements were performed in triplicates, and the entire experiment was repeated at least twice.

#### TEM of D-peptides

The D-peptides (ANK6, DB3DB3 and D3) were dissolved in ultra-pure water to 5 mM and incubated for 2–3 days at 37ºC with 300 rpm shaking. TEM grids were prepared and visualized as described above.

#### Thioflavin T kinetic assay of CsgA spine segments in the presence of the D-peptides

CsgA segments capped at the C-terminus were dissolved in DMSO to 10 mM stocks and stored at -20ºC until use. The peptides were thawed and diluted directly into a reaction buffer comprised of 50 mM potassium phosphate buffer, pH 7.3, mixed with filtered ThT from stock made in ultra-pure water, and with D-peptides from stock made in ultra-pure water, to the indicated final concentrations. The final concentration of ThT was 20 μM, in a final volume of 100 μL per well. The reaction was conducted as described above. The measurements were performed in triplicates, and the entire experiment was repeated at least three times.

#### Thioflavin T kinetic assay of PSMα3 with D-peptides

Fresh ThT reaction solution was prepared by a 10-fold dilution of a filtered ThT stock, made in ultra-pure water, into a reaction buffer containing 10 mM sodium phosphate buffer and 150 mM NaCl, pH 8.0. Lyophilized PSMα3 powder was freshly dissolved in TFA-HFIP (1:1), to a concentration of 5 mg/ml, sonicated for 3–5 min in a sonication bath, and evaporated using SpeedVac. The pretreated peptide was dissolved, on ice, to 1 mM in ultra-pure water, and sonicated on ice, for 3–5 min. The peptide was then diluted, on ice, with a filtered reaction buffer, and centrifuged for 5 min at 4°C and 10,000 rpm. The D-peptides DB3DB3 and ANK6 were each dissolved in ultra-pure water to a 50 mM stock concentration and stored at -80°C until use. The reaction was carried out in a Greiner bio-one black 96 well flat-bottom plate. For each reaction well, the supernatant of the PSMα3 solution was separately supplemented with the D-peptides in 1:5 PSMα3:D-peptide molar ratios, and with ThT to final concentrations of 50 μM PSMα3, 200 μM ThT and 250 μM D-peptide. The blank sample contained ThT mixed with the reaction buffer alone, or 250 μM of each of the D-peptides separately in the reaction buffer. The reaction plate was immediately covered with a silicone sealing film (ThermalSeal RTS), and incubated in a plate reader (CLARIOstar) at 37ºC, with 500 rpm shaking, for 15 sec before each cycle, with up to 1000 cycles of 5 min each. Measurements were made in triplicates. ThT fluorescence at an excitation of 438±20 nm and emission of 490±20 nm, was recorded. All triplicate values were averaged, appropriate blanks were subtracted, and the resulting values were plotted against time. Calculated standard errors of the mean are presented as error bars.

### CsgA fibrillation inhibition tested by SDS-PAGE migration

CsgA fibrils are insoluble in sodium dodecyl sulfate (SDS), even after boiling, and are thus unable to migrate in SDS polyacrylamide gel electrophoresis (SDS-PAGE), in contrast to soluble CsgA [71]. Therefore, this method can be used to monitor CsgA fibrillation and test potential inhibitors. Freshly purified recombinant CsgA was diluted in 50 mM potassium phosphate buffer, pH 7.3, mixed with diluted D-peptides into 1 mL tubes. Final concentrations used were 10 μM CsgA and 50 μM D-peptides (1:5 molar ratio). Samples were then incubated for 24 h at 25ºC, with 300 rpm shaking. Samples (20 μL) were then mixed with 10 μL 3× SDS sample buffer supplemented with dithiothreitol (DTT), and incubated at 95°C for 10 min. Samples were loaded on a 15% SDS-PAGE gel, and a known concentration of BSA solution was added to each lane to quantify the amount of protein in each band. Coomassie Stain (Expedeon—InstantBlue Coomassie Protein Stain) staining was performed to visualize the migration of soluble CsgA in the gel. Imaging was performed using a gel documentation system (Gel Doc–BioRad).

### Circular dichroism

Circular dichroism (CD) was used to monitor the secondary structure transitions of CsgA, in the presence or absence of the D-peptide inhibitor ANK6. Immediately prior to the CD experiment, freshly purified recombinant CsgA (in 50 mM potassium phosphate buffer, pH 7.3) was dialyzed at 4°C, using a Zeba spin 7K desalting column (ThermoFisher Scientific), into 2 mM potassium phosphate buffer, pH 7.3, to reduce background signal during CD measurements. CsgA was directly diluted into the CD cuvette to a final concentration of 9 μM. In parallel, 9 μM CsgA was mixed in a second cuvette with 45 μM ANK6 (1:5 molar ratio) diluted from a 50 mM stock, prepared in ultra-pure water. The signal from blank solutions of either 2 mM potassium phosphate buffer pH 7.3 alone, or of the same buffer containing 45 μM of ANK6, were recorded just before the addition of CsgA into the appropriate cuvette. CD measurements were performed at several time point over 18 h, with cuvettes being incubated at RT between measurements, and mixed thoroughly before each measurement.

Far UV CD spectra were recorded on the Applied Photophysics PiStar CD spectrometer (Surrey, UK) equilibrated with nitrogen gas, using a 0.1 mm path-length demountable quartz cell (Starna Scientific, UK). Changes in ellipticity were followed from 290 nm to 180 nm, with a 1-nm step size and a bandwidth of 1 nm. The measurements shown are an average of three scans for each time point, or two scans for blanks, captured at a scan rate of 1 sec per point.

### Attenuated total internal reflections Fourier transform infrared spectroscopy

Freshly purified recombinant CsgA in 50 mM potassium phosphate buffer, pH 7.3, was frozen in liquid nitrogen and lyophilized overnight to complete dryness. The dry CsgA powder was dissolved in deuterium oxide (D_2_O) to remove water residues interfering with the FTIR signal, and further lyophilized. This procedure was repeated twice. Immediately prior to measurements, the treated sample was dissolved in D_2_O to 20 mg/ml. Samples (5 μl) were spread on the surface of the ATR module (MIRacle Diamond w/ZnSe lens 3-Reflection HATR Plate; Pike Technologies) and allowed to dry under nitrogen gas. Absorption spectra were recorded using a Tensor 27 FTIR spectrometer (Bruker Optics). Measurements were performed in the wavelength range of 1500–1800 cm^-1^, in 2 cm^-1^ steps, and averaged over 100 scans. Background (ATR crystal) and blank (D_2_O) were measured and subtracted from the final spectra. The amide I’ region of the spectra (1600–1700 cm^-1^) is presented in the graphs.

### Crystallization of CsgA segments

Peptides synthesized with free (unmodified) termini were used for crystallization experiments to facilitate crystal contacts. _45_LNIYQY_50_ and _129_TASNSS_134_ formed crystals that diffracted well only when mixed with the _134_TAIVVQ_139_ segment from the R5 repeat of the nucleator protein CsgB prior to crystallization. TAIVVQ was not present in the crystals but aided in crystallization. It is possible that this phenomenon is relevant to the mechanism of heteronucleation of CsgA by CsgB in-vivo [5]. IYQYGG (10 mM) was dissolved in water. VTQVGF (10 mM) was dissolved in 100% DMSO. LNIYQY crystals were grown in a mixture of 10 mM LNIYQY and 10 mM TAIVVQ dissolved in 82% DMSO. TASNSS crystals were grown in a mixture of 30 mM TASNSS and 10 mM TAIVVQ, dissolved in 82% DMSO. Peptide solution drops (100 nL) were dispensed onto crystallization screening plates, using the Mosquito automated liquid dispensing robot (TTP Labtech, UK) located at the Technion Center for Structural Biology (TCSB). Crystallization using the hanging drop method, was performed in 96-well plates, with 100 μL solution in each well. The drop volumes were 150–300 nL. All plates were incubated in a Rock imager 1000 robot (Formulatrix), at 293 K. Micro-crystals grew after few days and were mounted on glass needles glued to brass pins [130]. No cryo protection was used. Crystals were kept at RT prior to data collection. Structures were obtained from drops that were a mixture of the following peptide and reservoir solutions: IYQYGG: 10 mM IYQYGG, 0.1 M sodium acetate, pH 4.6, and 2.0 M sodium formate. LNIYQY: 10 mM LNIYQY, 10 mM TAIVVQ, 0.1 M HEPES, pH 7.5, and 20%v/v Jeffamine M-600. VTQVGF: 10 mM VTQVGF, 3.0 M sodium chloride, 0.1 M BIS-Tris, pH 5.5. TASNSS: 30 mM TASNSS, 10 mM TAIVVQ, 0.2 M lithium sulfate, 0.1 M Tris-HCl, pH 8.5, and 30% (w/v) polyethylene glycol 4000.

### Structure determination and refinement

X-ray diffraction data were collected at 100 K, using 5˚ oscillation. The X-ray diffraction data for VTQVGF were collected at the micro-focus beamline ID23-EH2 of the European Synchrotron Radiation Facility (ESRF) in Grenoble, France; wavelength of data collection was 0.8729 Å. The X-ray diffraction data for LNIYQY, IYQYGG and TASNSS were collected at the micro-focus beamline P14 operated by EMBL at the PETRAIII storage ring (DESY, Hamburg, Germany); wavelength of data collection was 0.9763 Å. Data indexation, integration and scaling were performed using XDS/XSCALE [131]. Molecular replacement solutions for all segments were obtained using the program Phaser [132] within the CCP4 suite [132–134]. The search models consisted of geometrically idealized β-strands. Crystallographic refinements were performed with the program Refmac5 [134]. Model building was performed with Coot [135] and illustrated with Chimera [136]. There were no residues that fell in the disallowed region of the Ramachandran plot. Crystallographic statistics are listed in S2 Table.

### Calculations of structural properties

The Lawrence and Colman’s shape complementarity index [137] was used to calculate the shape complementarity between pairs of sheets forming the dry interface (S3 Table). The buried surface area was calculated with Chimera (UCSF), with a default probe radius and vertex density of 1.4 Å and 2.0/Å^2^, respectively. The number of solvent accessible buried surface areas was calculated as the average area buried of one strand within two β-sheets (total area buried from both sides is double the reported number in S3 Table).

### Bacterial strains and growth conditions

*Salmonella typhimurium* bacterial strains used: the MAE52 strain displays a constitutive multicellular morphotype mediated by the expression of the *agfD* operon, leading to the production of an adhesive extracellular matrix consisting of curli fimbriae (previously called thin aggregative fimbriae (*agf*)) and cellulose [138]. The MAE150 (Δ*bcsA*) strain sustained a deletion of the bacterial cellulose synthesis (*bcs*), a gene encoding cellulose synthase [139]. *S*. *typhimurium* bacterial MAE52 and MAE150 strains were grown on LB agar plates without NaCl, at 30°C. For liquid media growth, a single bacterial colony was picked from an agar plate and dipped in LB medium (lacking NaCl), followed by incubation at 30°C with vigorous shaking.

### Static biofilm analysis

The evaluation of static biofilm production was adapted from a previous study [140]. Bacterial suspensions of *S*. *typhimurium* MAE52, at OD_600_ of 0.05–0.13, were incubated in 200 μl LB medium containing D-peptide inhibitors (0, 5, 10, 20, or 40 μM) for 48 h, at 30°C, in a 96-well plate (nunclon, Roskilde, Denmark). The plate was then washed twice with 0.9% saline (w/v).

#### Surface-attached biofilm assay

The protocol was adapted from previous reports [141, 142]. Specifically, after washing the plates, biofilm cells that remained attached to the bottom of the micro-wells were fixated with 4% paraformaldehyde (w/v), washed tree times with 0.9% saline (w/v), stained with propidium iodide and analyzed using a Zeiss 710 confocal laser scanning microscope (CLSM, Jena, Germany), with 10 x lenses and 522 nm and 682 nm excitation/emission filters.

#### Total biofilm biomass assay

In a separated experiment, pellicle biofilms attached to the sides of the micro-wells, at the liquid-air interface, as well as surface-attached biofilms, were analyzed using standard crystal violet staining. After washing the wells, as described above in the surface-attached biofilm assay, 0.1% crystal violet staining solution (200 μl) was added to each well and incubated for 15 min, followed by removal of the dye and washing with 0.9% saline. Ethanol (95%; 200 μl) was added to each micro-well and incubated for 30 min, after which, 100 μl from each well were transferred to a new 96-micro-well plate; OD_600_ was recorded with a Synthergi HT (Biotek, Winooski, USA) device.

### Curli production analysis using congo red staining

Curli (along with cellulose) production is associated with CR staining in *S*. *typhimurium* [73], thus the lack of CR adsorption by the bacterial cells is evident in the residual agar below the colony [8]. *S*. *typhimurium* MAE150 was grown overnight in LB medium and then diluted to obtain a bacterial suspension of OD_600_ of 0.05–0.13. D-peptide inhibitors (0, 10, 20, or 40 μM) were added to the medium and 3 μl of the suspension was spotted on a LB-agar plate supplemented with 20 μg/ml CR. The bacterial biofilm colonies were allowed to grow for 48 h at 30°C, after which, the colony was immersed in 0.4% paraformaldehyde, followed by the removal of the colony with double distilled water washes. In order to measure the amount of CR that remained on the agar after the removal of the colonies, the agar was dissolved as previously described [140]. In brief, the agar underneath the removed colony was cut out, and incubated at 55°C for 10 min, in 1 ml of binding buffer solution (Hylabs, Rehovot, Israel). The residual CR in the melted agar was measured by absorbance at OD_510_ (optical density was calibrated after scanning the optimum wave length for CR detection), using nanodrop 2000c (Thermo).

### Statistical analysis

Statistical analyses were carried out for the total biofilm biomass assay (crystal violet staining) and for curli production analysis (CR staining). The data were not normally distributed according to the Shapiro Wilk test. The analyses were performed using the non-parametric Kruskal-Wallis test for comparing three independent groups and the Mann-Whitney non-parametric test for comparing two independent groups (all variables calculated had a small sample size (N ≥10)). All tests applied were two-tailed, and a p-value of 5% or less was considered statistically significant. Analyses were performed using SPSS software, version 24.

## Supporting information

S1 Fig Electron micrographs of CsgA segments and the D-peptide inhibitors. **A**. TEM micrographs visualizing fibrils of _45_LNIYQY_50_ (R1), _47_IYQYGG_52_ (R1), _137_VTQVGF_142_ (R5) and _129_TASNSS_134_ (R4-R5 loop). Scale bars are indicated. **B**. TEM micrographs of incubated D-peptide inhibitors.
(TIF)

S2 Fig Concentration-dependent ThT fibrillation kinetics of CsgA segments. The graphs represent averaged fluorescence reading of ThT triplicated measurements of the CsgA segments at 100, 150 and 200 μM. Error bars represent standard error of the mean calculated from a triplicate.
(TIF)

S3 Fig Structural description of the _45_LNIYQY_50_ fibril. The crystal structure of _45_LNIYQY_50_ demonstrates the formation of a cross-β steric zipper fibril composed of mated, parallel β-sheets. Two possible tight and dry interfaces are observed in the crystal. The first dry interface between mated β-sheets is mostly hydrophobic, formed between facing and tightly packed Leu45 and Ile47 residues flanked by Gln49 side chains. In this conformation, water molecules running along the fibril axis may form hydrogen bonds with the Gln49 side chains as well as with the C-terminus carboxyl group. The second interface is predominantly mediated by two tyrosine residues (Tyr48 and Tyr50). These tyrosine residues face each other, forming a tight and dry interface along the fibril axis. Tyr50 from each strand may form hydrogen bonds with equivalent tyrosines from facing and adjacent strands, creating a network of hydrogen bonds within the dry interface along the fibril axis. The Asn46 residues are facing the same direction as the tyrosines on the β-strands, but do not directly participate in the interface between mating sheets. However, these asparagine residues putatively form a ladder of hydrogen bonds along the fibril axis (not shown), further stabilizing the fibril structure. The carbons of each β-sheet are colored either gray or purple; heteroatoms are colored by atom type (nitrogen in blue, oxygen in red). Water molecules are shown as small cyan spheres. Hydrogen bonds are shown in cyan lines.
(TIF)

S4 Fig Structural description of the _47_IYQYGG_52_ fibril. The _47_IYQYGG_52_ segment, which partially overlaps with _45_LNIYQY_50_, also forms two possible dry zipper interfaces. The first interface is mediated via Ile47, Gln49, and Gly51 from both sides of the mated β-sheets. Each Gln49, located in the middle of the interface, may participate in hydrogen bonds with adjacent glutamines along the sheet (not shown) and with the backbone oxygen of Tyr50. As with _45_LNIYQY_50_, the second interface is mediated by Tyr48 and Tyr50. However, in _47_IYQYGG_52_, Tyr48 from each strand forms hydrogen bonds with equivalent tyrosines from facing and adjacent strands, creating a network of hydrogen bonds within the dry interface along the fibril axis. Water molecules flank the dry interface, putatively engaging in hydrogen bonds with Tyr50, with the C-terminus carboxyl group, and with the N-terminal amine group along the fibril axis. Coloring scheme is as in S3 Fig.
(TIF)

S5 Fig Structural description of the _137_VTQVGF_142_ fibril. The crystal structure of _137_VTQVGF_142_ shows two possible dry interfaces between parallel mated β-sheets. One interface is mediated by Thr138, Val140, and Phe142. These residues are tightly packed forming a hydrophobic, dry, interface, with the side chain oxygen of Thr138 positioned at the periphery of the interface, forming putative hydrogen bonds with water molecules along the fibril axis. The second dry interface is mediated via Val137, Gln139, and Gly141. As with _47_IYQYGG_52,_ the glutamines are located in the middle of the interface and engage in putative hydrogen bonds with adjacent glutamines along the sheet (not shown) as well as with backbone oxygens, here of Val140. Coloring scheme is as in S3 Fig.
(TIF)

S6 Fig Structural description of the _129_TASNSS_134_ fibril. _129_TASNSS_134_ from the R4-R5 loop region was selected as a control sequence. This segment was predicted by computational methods to be amyloidogenic but is located in a region not implicated in fibrillation. In contrast to the other three segments that form tightly packed steric zipper structures, the _129_TASNSS_134_ segment forms extended chains yielding anti-parallel β-sheets. Each β-sheet is composed of anti-parallel strands putatively stabilized within the sheet both by hydrogen bonds between backbone atoms along the sheets as well as electrostatic interactions between the C- and N-termini. Furthermore, the C-terminal Ser134 can form hydrogen bonds with the N-termini of adjacent strands on the same sheet. In contrast to the other three spine segments from the R1 and R5 repeats, the β-sheets of _129_TASNSS_134_ do not mate via a tight interface. Each sheet is not directly facing another sheet but shifted. Nevertheless, several inter-sheet interactions stabilize this configuration, including possible hydrogen bonds between Thr129 and Ser133, Ser134 and the backbone oxygen of Asn132, and Ser131 and the N-terminus (bonds not shown due to antiparallel orientation that prevents a clear visualization). This architecture is chemically stable though it does not strictly belong to a class of steric zippers. In accordance with its unusual structure, this segment forms ribbon-like structures with atypical morphology as demonstrated by TEM (S1 Fig). These atypical ribbons do not bind ThT (S2 Fig). Coloring scheme is as in S3 Fig.
(TIF)

S7 Fig ATR-FTIR spectra demonstrates the cross-β architecture of full-length CsgA fibrils. Attenuated total internal reflection Fourier transform infrared (ATR-FTIR) spectroscopy of the amide I’ region (1600–1700 cm^-1^) of CsgA fibrils shows a main peak at 1617 cm^-1^ corresponding to rigid amyloid fibrils [67–69]. The black line represents the ATR spectra and the red line is the second derivative.
(TIF)

S8 Fig Fibril seeds of CsgA and segments enhance fibrillation of Aβ_1–40_. **A.** ThT measurements of 50 μM Aβ_1–40_ alone and with the addition of seeds of CsgA fibrils at 1 or 5% vol. **B**. TEM micrographs of CsgA fibril seeds at 1% vol, Aβ_1–40_ incubated alone, and Aβ_1–40_ incubated in the presence of CsgA fibril seeds at 1% vol showing massive fibrillation. Scale bars of 300 nm are indicated. **C**. ThT measurements of 50 μM Aβ_1–40_ alone and with the addition of fibril seeds of CsgA segments at 0.2 or 1% vol. **D.** ThT measurements of only the fibril seeds of CsgA and the segments, which correspond to the measurements shown in **A&C**, showing minimal flouresence except from LNIYQY at 1% vol that shows elevated ThT fluorescence. In (**A**, **C & D**), error bars represent standard error of the mean calculated from triplicate measurements. Each graph represents three independent experiments repeated on different days.
(TIF)

S9 Fig D-peptides inhibit fibrillation of CsgA in dose-dependent manners. The graphs show mean fluorescence readings of triplicate ThT measurements of CsgA with or without ANK6 (A), DB3DB3 (B) or D3 (C) at different molar ratios as shown in the color-coded bar. Error bars represent standard error of the mean calculated from triplicates. The D-peptides delayed fibril formation of CsgA and reduced the fluorescence signal in dose-dependent manners.
(TIF)

S10 Fig D-peptides inhibit fibrillation of CsgA spine segments. The graphs show mean fluorescence readings of triplicate ThT measurements of CsgA spine segments in the presence of the D-peptide inhibitors. Fibrillation of 100μM _45_LNIYQY_50_ was assessed with 0, 100, 200 and 500 μM ANK6 (**A**), DB3DB3 (**B**) or D3 (**C**) peptides. (**D**) Fibrillation of 500μM _137_VTQVGF_142 at_ was examined with 500 μM of ANK6, DB3DB3 or D3 peptides. Error bars represent standard error of the mean calculated from triplicates. Each graph represents at least three independent experiments.
(TIF)

S11 Fig ANK6 prevents secondary structure transitions of CsgA. Time-dependent CD spectra of CsgA incubated alone (**A**) or in the presence of ANK6 (**B**) (1:5 molar ratio). The changes in ellipticity are shown along a wavelength range of 190–265 nm.
(TIF)

S12 Fig The D-peptides prolong soluble state of CsgA. Migration of soluble CsgA detected by Coomassie-blue staining of 15% SDS-PAGE gel. Incubated CsgA forms insoluble fibrils and does not migrate on the gel compared to freshly purified CsgA. CsgA incubated with ANK6 and DB3DB3 at 1:5 molar ratios showed a prolonged soluble state of CsgA, indicating inhibition of the formation of insoluble fibrils.
(TIF)

S13 Fig SEC-MALS analysis of freshly purified CsgA. SEC-MALS chromatogram of CsgA presents two main populations with different molecular weights. The major peak corresponds to monomeric CsgA, while the minor peak corresponds to hexamers of CsgA.
(TIF)

S14 Fig Effect of D-peptides on the growth of *S*. *typhimurium*. Bacterial growth of the MAE52 strain was assessed in the presence of the D-peptides at concentrations of 0, 75, 150 and 500 μM by a spectrophotometer at OD_600_. An average of triplicate readings was recorded every 20 minutes. No significant effect on the growth phase was evident except with 500 μM of ANK6.
(TIF)

S15 Fig Effect of D-peptides on *S*. *typhimurium* biofilm grown on CR-supplemented agar. (**A**) *S*. *typhimurium* MAE52 strain grown on plates with CR-supplemented agar for 48 h at 30°C show reddish biofilm colony that adsorbed the dye (left colony on the image). The addition of ANK6 at 20 μM (middle colony) or 40 μM (right colony) show a dose-dependent discoloration at the center of the colony where the drop of the bacteria and D-peptide suspension was placed, indicating less CR adsorption. (**B**) *S*. *typhimurium* MAE150 strain (cellulose deficient mutant) colonies were grown on plates with CR-supplemented agar for 48 h at 30 °C; the images depict residual stain on the agar following the removal of the biofilm colonies. From left to right: ANK6, DB3DB3 and D3 added at different concentrations in a clockwise manner, starting in the upper-right corner (0, 10, 20 and 40 μM). Each plate represents a triplicate of repeats and the phenomenon was exhibited in three independent experiments. Quantification of the residual stain is shown in Fig 6.
(TIF)

S1 Table Sequences of CsgA repeats and of the D-peptide inhibitors. (DOCX)

S2 Table Data collection and refinement statistics (molecular replacement). (DOCX)

S3 Table Features of the CsgA spine structures compared to the NNQQNY steric zipper structure. (DOCX)

S1 References References that appear in supporting information tables. (DOCX)
