# Supplementary figures and images for: Structural Insights into Curli CsgA Cross-β Fibril Architecture Inspire Repurposing of Anti-amyloid Compounds as Anti-biofilm Agents
Source: PLoS Pathog. 2019 Aug 30;15(8):e1007978. doi: 10.1371/journal.ppat.1007978 (PMC6748439; doi:10.1371/journal.ppat.1007978)

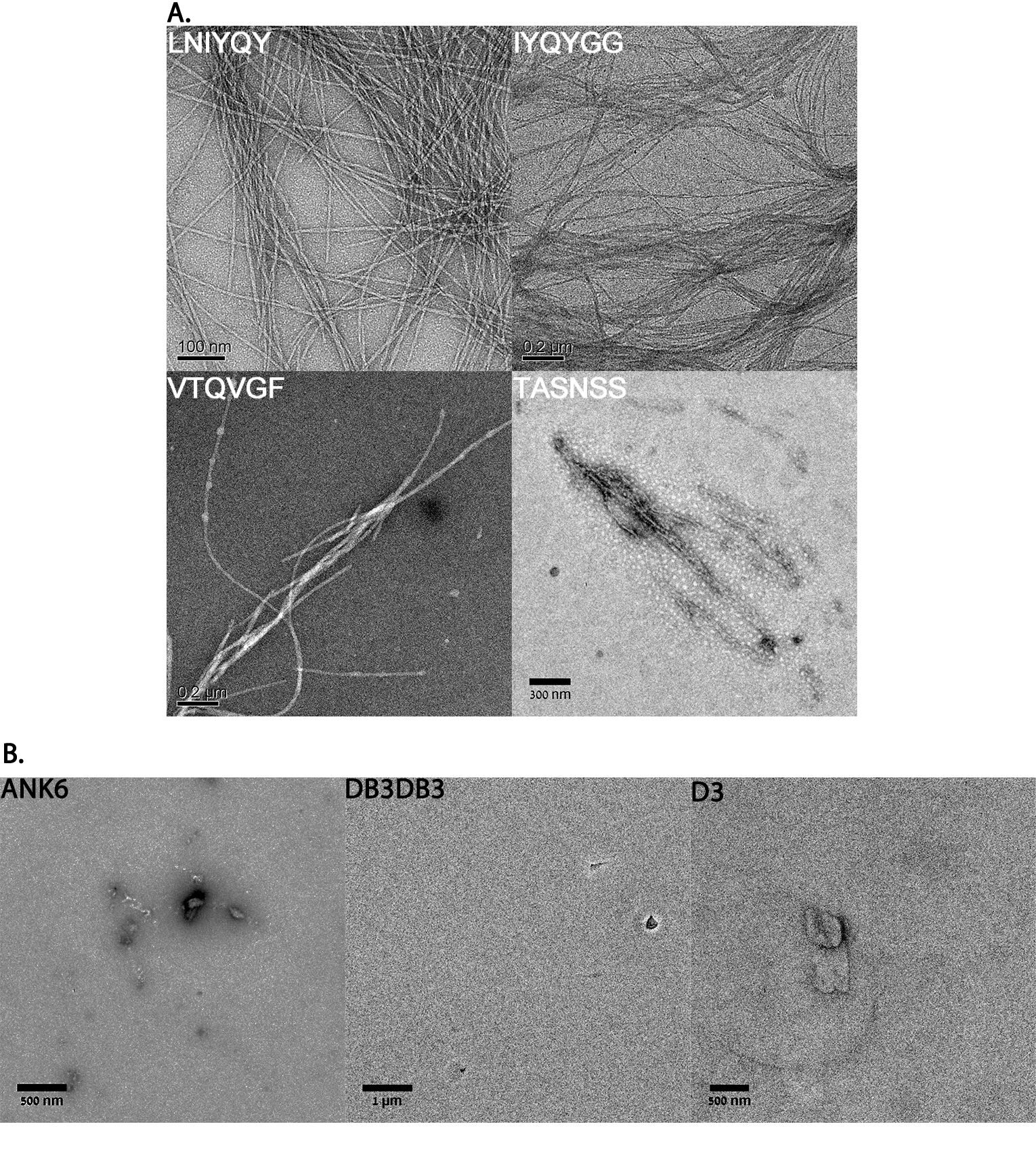

Supplement: S1 Fig — A. TEM micrographs visualizing fibrils of 45LNIYQY50 (R1), 47IYQYGG52 (R1), 137VTQVGF142 (R5) and 129TASNSS134 (R4-R5 loop). Scale bars are indicated. B. TEM micrographs of incubated D-peptide inhibitors. (TIF) [file ppat.1007978.s001.tif]

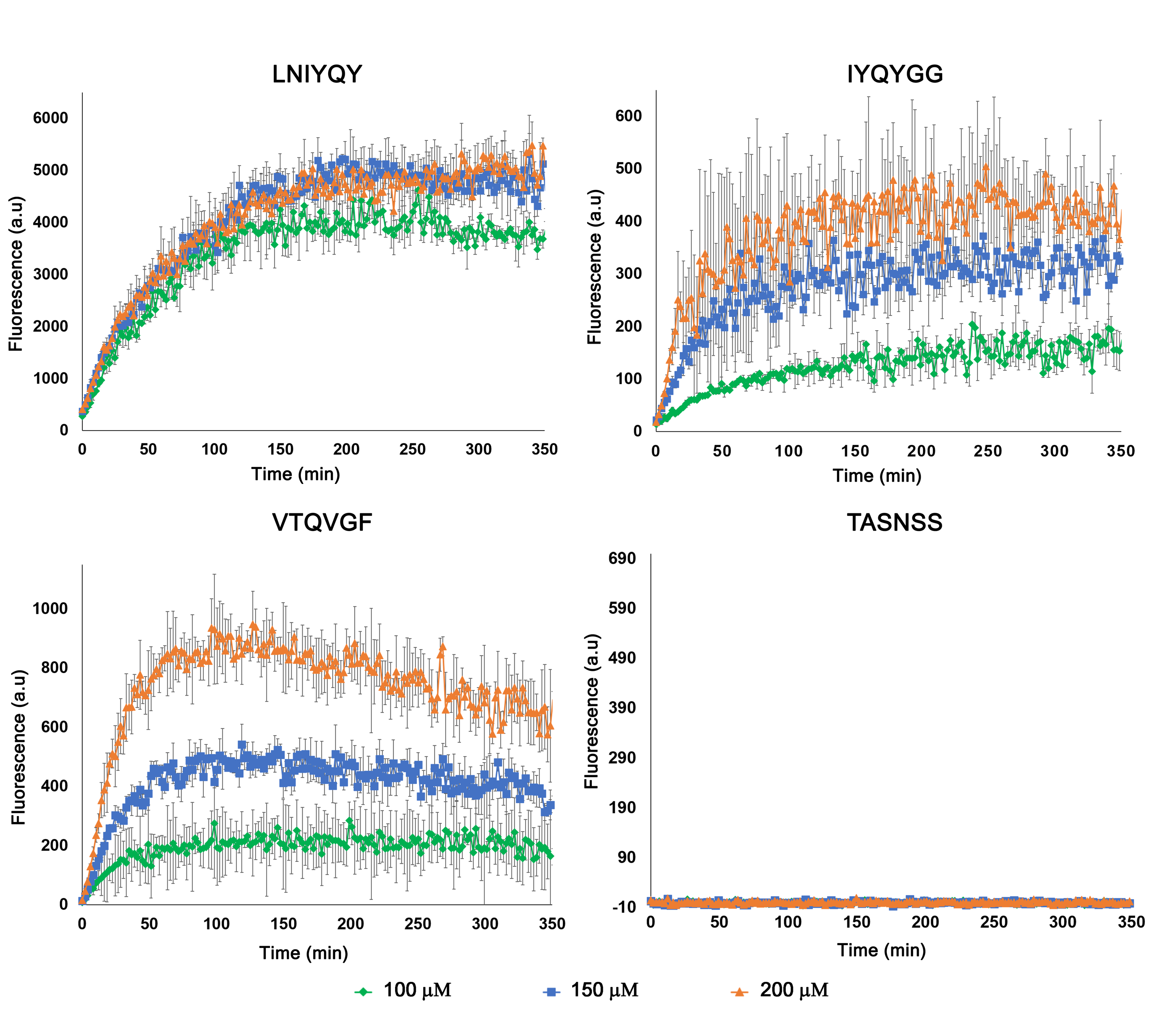

Supplement: S2 Fig — The graphs represent averaged fluorescence reading of ThT triplicated measurements of the CsgA segments at 100, 150 and 200 μM. Error bars represent standard error of the mean calculated from a triplicate. (TIF) [file ppat.1007978.s002.tif]

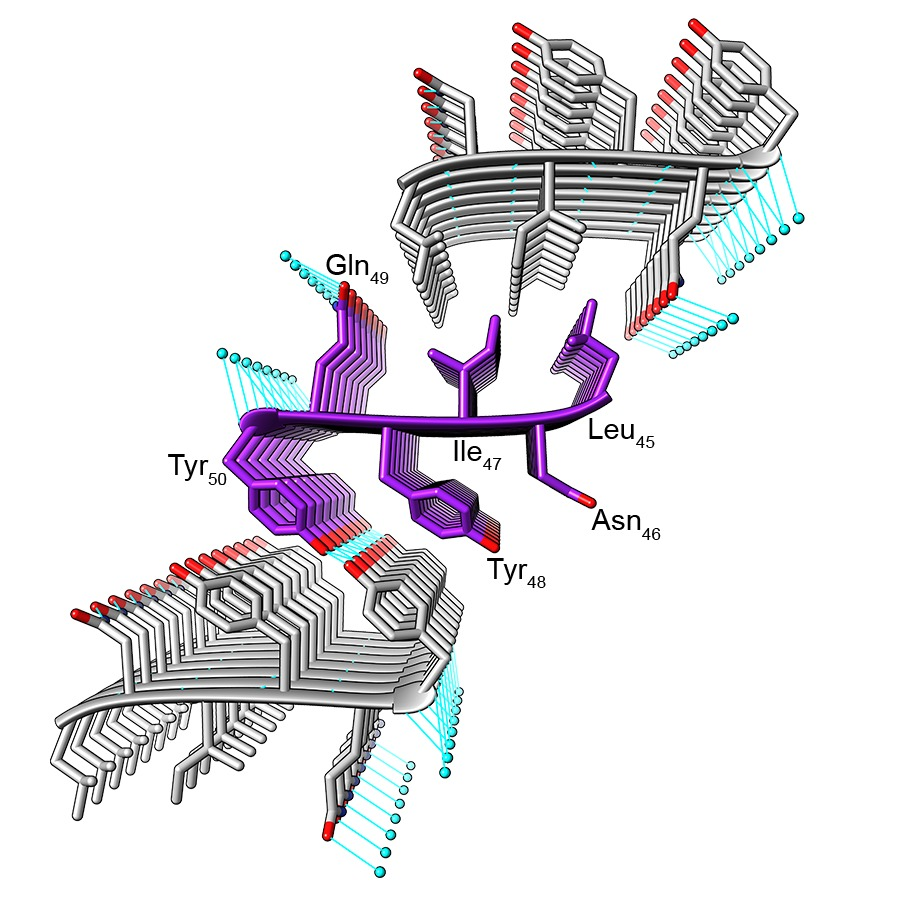

Supplement: S3 Fig — The crystal structure of 45LNIYQY50 demonstrates the formation of a cross-β steric zipper fibril composed of mated, parallel β-sheets. Two possible tight and dry interfaces are observed in the crystal. The first dry interface between mated β-sheets is mostly hydrophobic, formed between facing and tightly packed Leu45 and Ile47 residues flanked by Gln49 side chains. In this conformation, water molecules running along the fibril axis may form hydrogen bonds with the Gln49 side chains as well as with the C-terminus carboxyl group. The second interface is predominantly mediated by two tyrosine residues (Tyr48 and Tyr50). These tyrosine residues face each other, forming a tight and dry interface along the fibril axis. Tyr50 from each strand may form hydrogen bonds with equivalent tyrosines from facing and adjacent strands, creating a network of hydrogen bonds within the dry interface along the fibril axis. The Asn46 residues are facing the same direction as the tyrosines on the β-strands, but do not directly participate in the interface between mating sheets. However, these asparagine residues putatively form a ladder of hydrogen bonds along the fibril axis (not shown), further stabilizing the fibril structure. The carbons of each β-sheet are colored either gray or purple; heteroatoms are colored by atom type (nitrogen in blue, oxygen in red). Water molecules are shown as small cyan spheres. Hydrogen bonds are shown in cyan lines. (TIF) [file ppat.1007978.s003.tif]

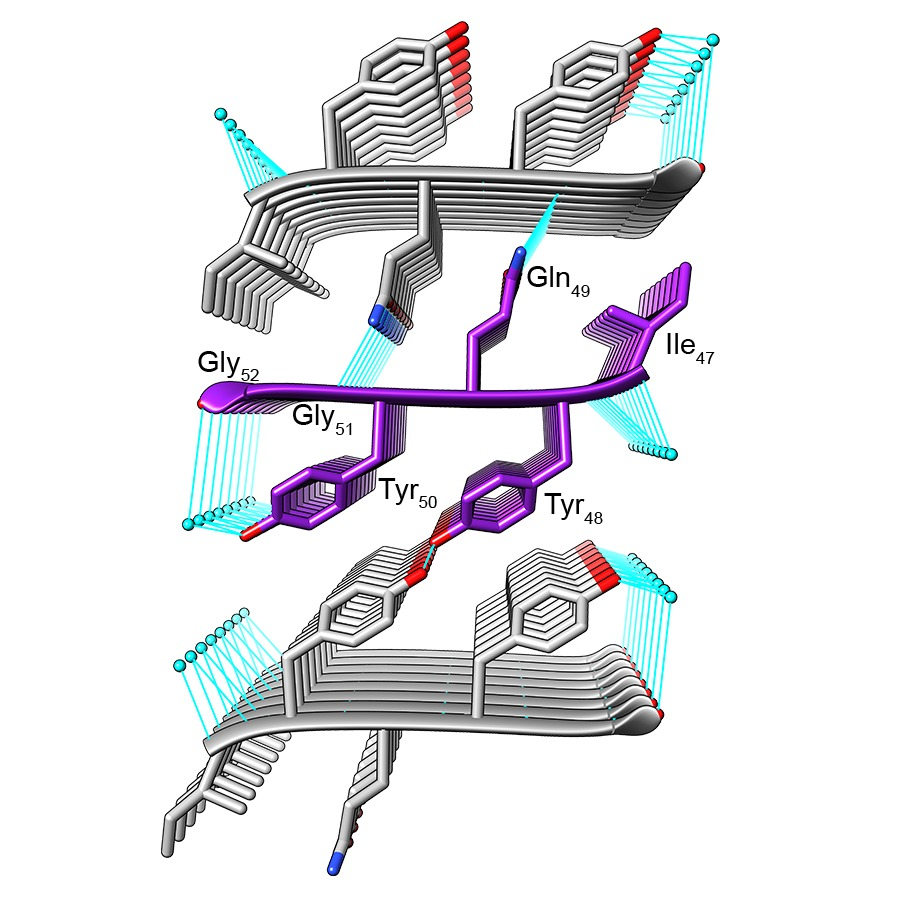

Supplement: S4 Fig — The 47IYQYGG52 segment, which partially overlaps with 45LNIYQY50, also forms two possible dry zipper interfaces. The first interface is mediated via Ile47, Gln49, and Gly51 from both sides of the mated β-sheets. Each Gln49, located in the middle of the interface, may participate in hydrogen bonds with adjacent glutamines along the sheet (not shown) and with the backbone oxygen of Tyr50. As with 45LNIYQY50, the second interface is mediated by Tyr48 and Tyr50. However, in 47IYQYGG52, Tyr48 from each strand forms hydrogen bonds with equivalent tyrosines from facing and adjacent strands, creating a network of hydrogen bonds within the dry interface along the fibril axis. Water molecules flank the dry interface, putatively engaging in hydrogen bonds with Tyr50, with the C-terminus carboxyl group, and with the N-terminal amine group along the fibril axis. Coloring scheme is as in S3 Fig. (TIF) [file ppat.1007978.s004.tif]

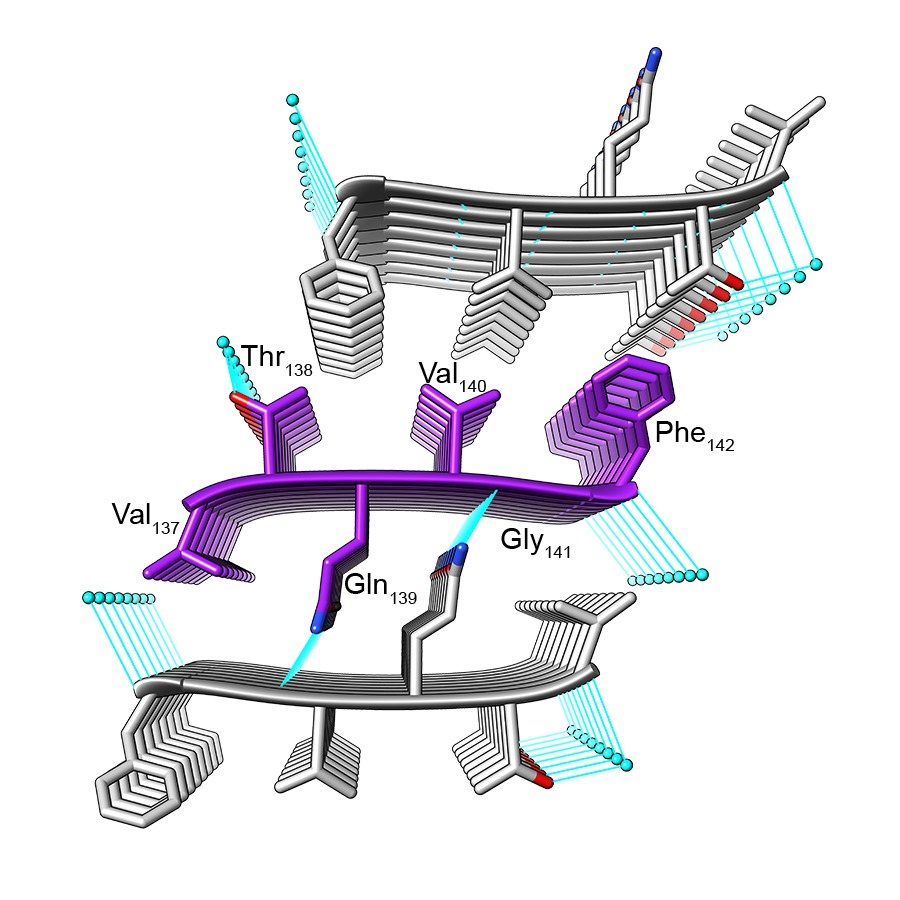

Supplement: S5 Fig — The crystal structure of 137VTQVGF142 shows two possible dry interfaces between parallel mated β-sheets. One interface is mediated by Thr138, Val140, and Phe142. These residues are tightly packed forming a hydrophobic, dry, interface, with the side chain oxygen of Thr138 positioned at the periphery of the interface, forming putative hydrogen bonds with water molecules along the fibril axis. The second dry interface is mediated via Val137, Gln139, and Gly141. As with 47IYQYGG52, the glutamines are located in the middle of the interface and engage in putative hydrogen bonds with adjacent glutamines along the sheet (not shown) as well as with backbone oxygens, here of Val140. Coloring scheme is as in S3 Fig. (TIF) [file ppat.1007978.s005.tif]

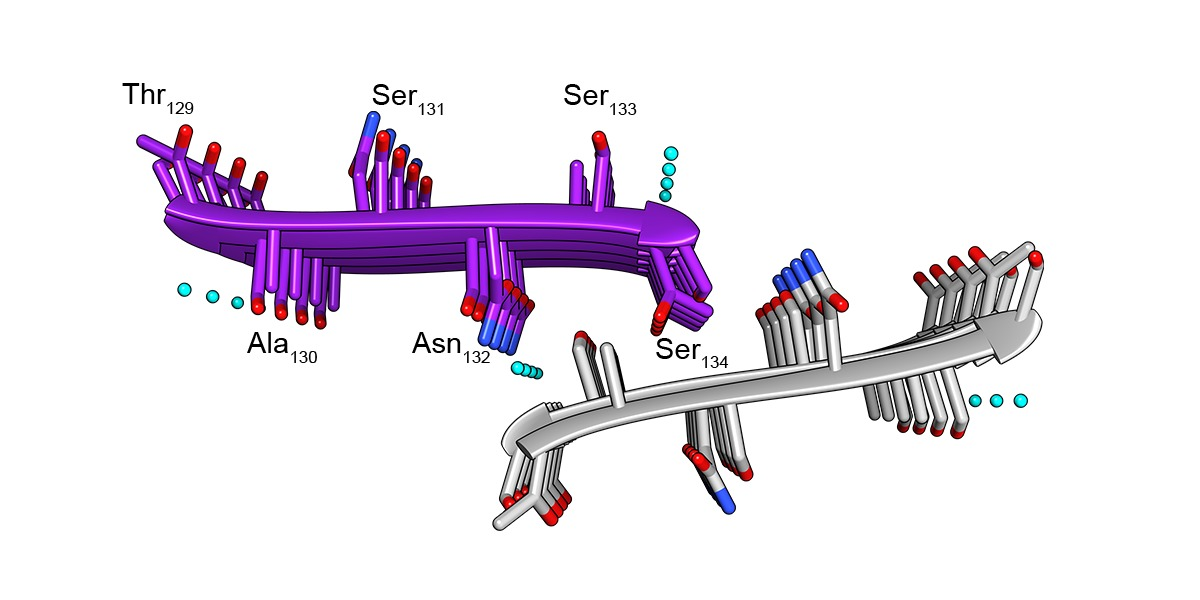

Supplement: S6 Fig — 129TASNSS134 from the R4-R5 loop region was selected as a control sequence. This segment was predicted by computational methods to be amyloidogenic but is located in a region not implicated in fibrillation. In contrast to the other three segments that form tightly packed steric zipper structures, the 129TASNSS134 segment forms extended chains yielding anti-parallel β-sheets. Each β-sheet is composed of anti-parallel strands putatively stabilized within the sheet both by hydrogen bonds between backbone atoms along the sheets as well as electrostatic interactions between the C- and N-termini. Furthermore, the C-terminal Ser134 can form hydrogen bonds with the N-termini of adjacent strands on the same sheet. In contrast to the other three spine segments from the R1 and R5 repeats, the β-sheets of 129TASNSS134 do not mate via a tight interface. Each sheet is not directly facing another sheet but shifted. Nevertheless, several inter-sheet interactions stabilize this configuration, including possible hydrogen bonds between Thr129 and Ser133, Ser134 and the backbone oxygen of Asn132, and Ser131 and the N-terminus (bonds not shown due to antiparallel orientation that prevents a clear visualization). This architecture is chemically stable though it does not strictly belong to a class of steric zippers. In accordance with its unusual structure, this segment forms ribbon-like structures with atypical morphology as demonstrated by TEM (S1 Fig). These atypical ribbons do not bind ThT (S2 Fig). Coloring scheme is as in S3 Fig. (TIF) [file ppat.1007978.s006.tif]

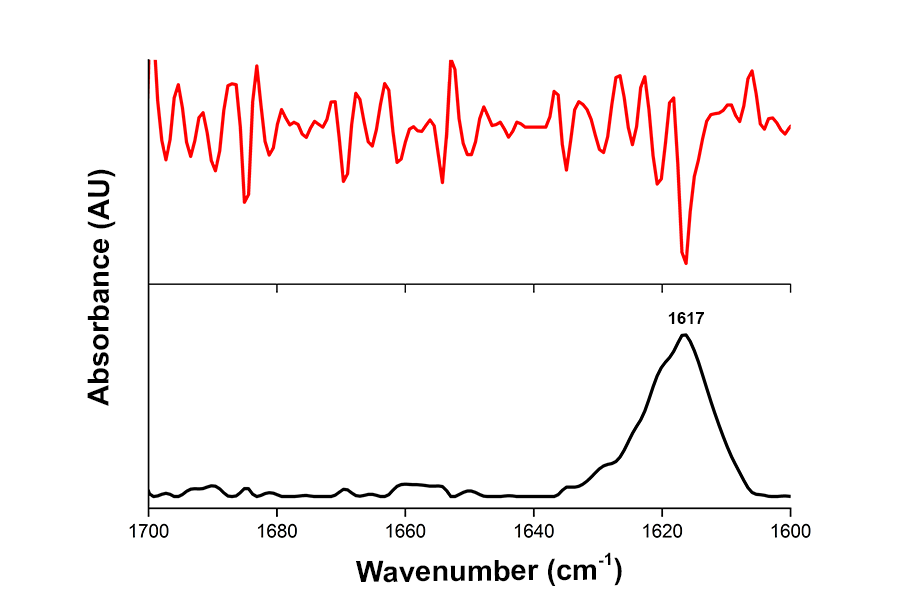

Supplement: S7 Fig — Attenuated total internal reflection Fourier transform infrared (ATR-FTIR) spectroscopy of the amide I’ region (1600–1700 cm-1) of CsgA fibrils shows a main peak at 1617 cm-1 corresponding to rigid amyloid fibrils [67–69]. The black line represents the ATR spectra and the red line is the second derivative. (TIF) [file ppat.1007978.s007.tif]

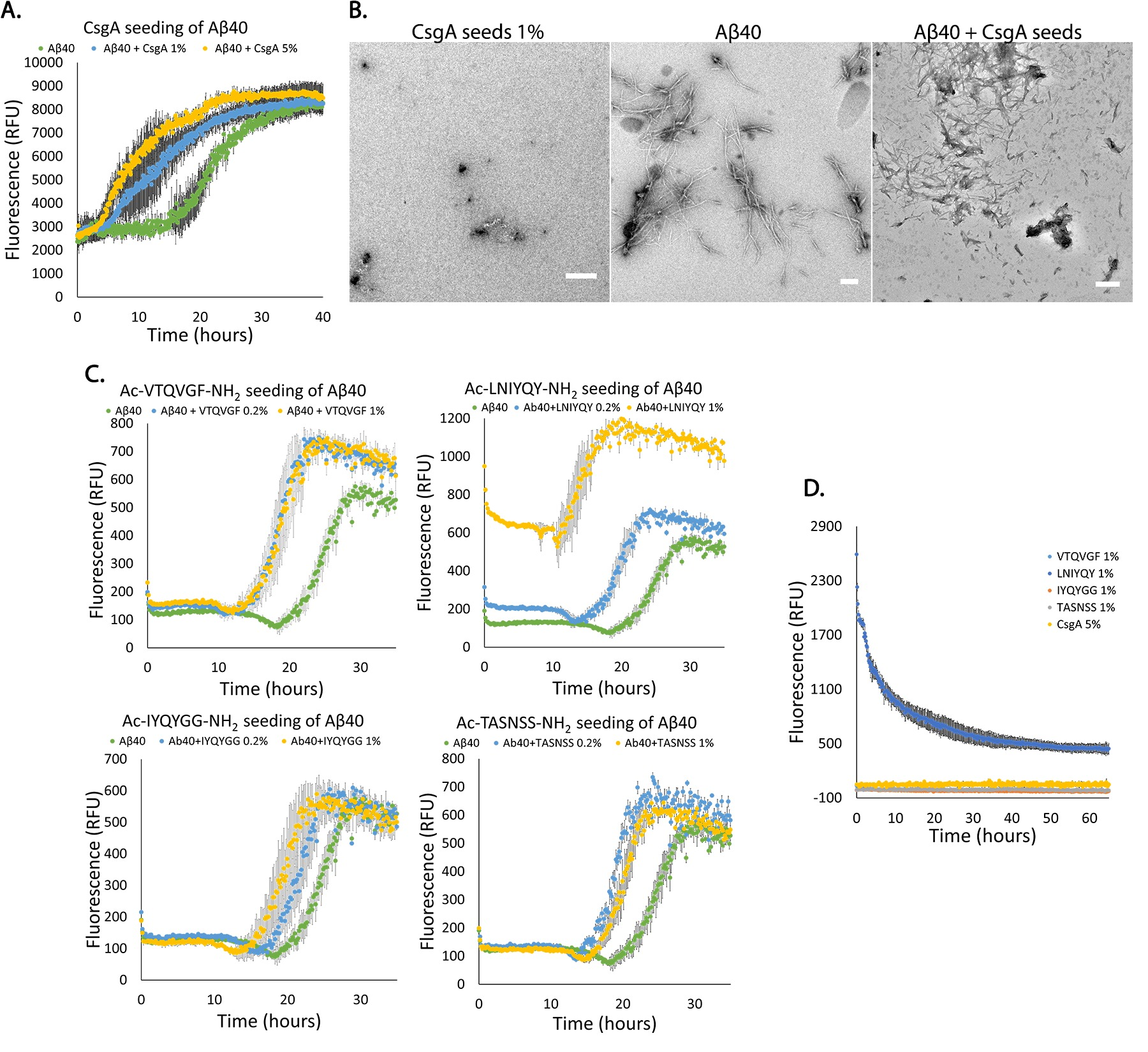

Supplement: S8 Fig — A. ThT measurements of 50 μM Aβ1–40 alone and with the addition of seeds of CsgA fibrils at 1 or 5% vol. B. TEM micrographs of CsgA fibril seeds at 1% vol, Aβ1–40 incubated alone, and Aβ1–40 incubated in the presence of CsgA fibril seeds at 1% vol showing massive fibrillation. Scale bars of 300 nm are indicated. C. ThT measurements of 50 μM Aβ1–40 alone and with the addition of fibril seeds of CsgA segments at 0.2 or 1% vol. D. ThT measurements of only the fibril seeds of CsgA and the segments, which correspond to the measurements shown in A&C, showing minimal flouresence except from LNIYQY at 1% vol that shows elevated ThT fluorescence. In (A, C & D), error bars represent standard error of the mean calculated from triplicate measurements. Each graph represents three independent experiments repeated on different days. (TIF) [file ppat.1007978.s008.tif]

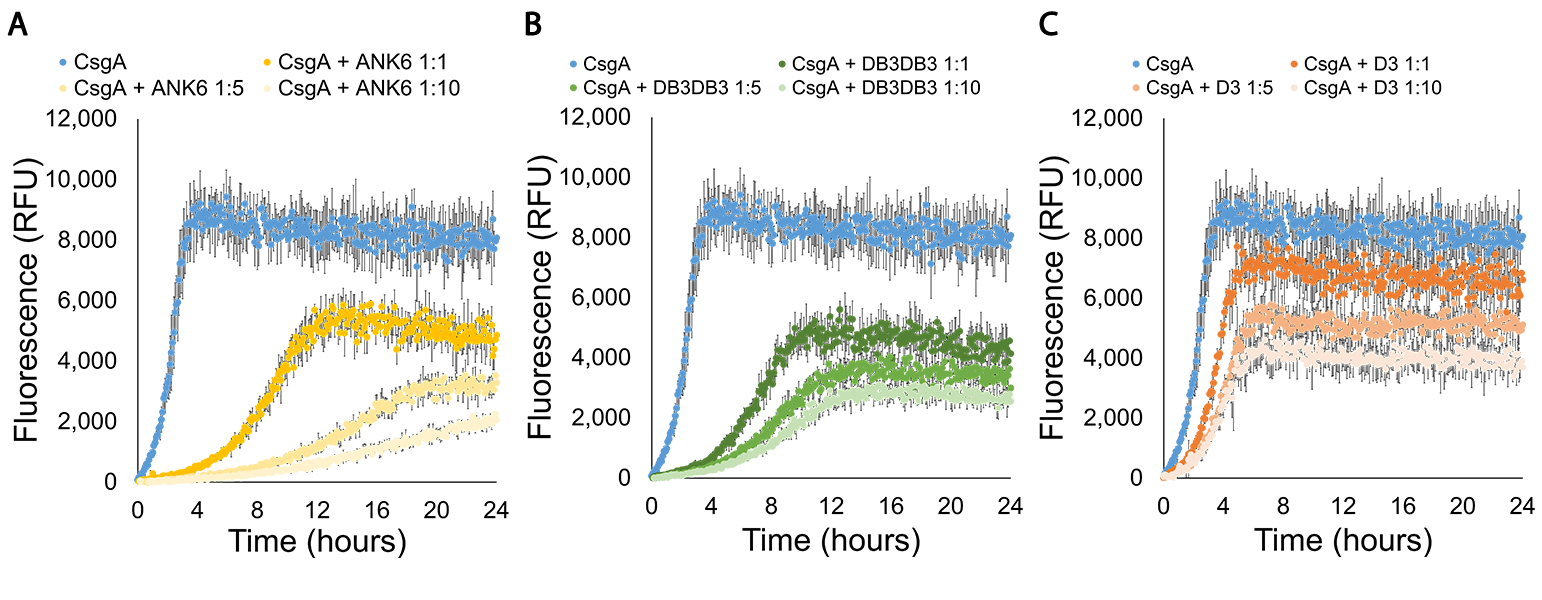

Supplement: S9 Fig — The graphs show mean fluorescence readings of triplicate ThT measurements of CsgA with or without ANK6 (A), DB3DB3 (B) or D3 (C) at different molar ratios as shown in the color-coded bar. Error bars represent standard error of the mean calculated from triplicates. The D-peptides delayed fibril formation of CsgA and reduced the fluorescence signal in dose-dependent manners. (TIF) [file ppat.1007978.s009.tif]

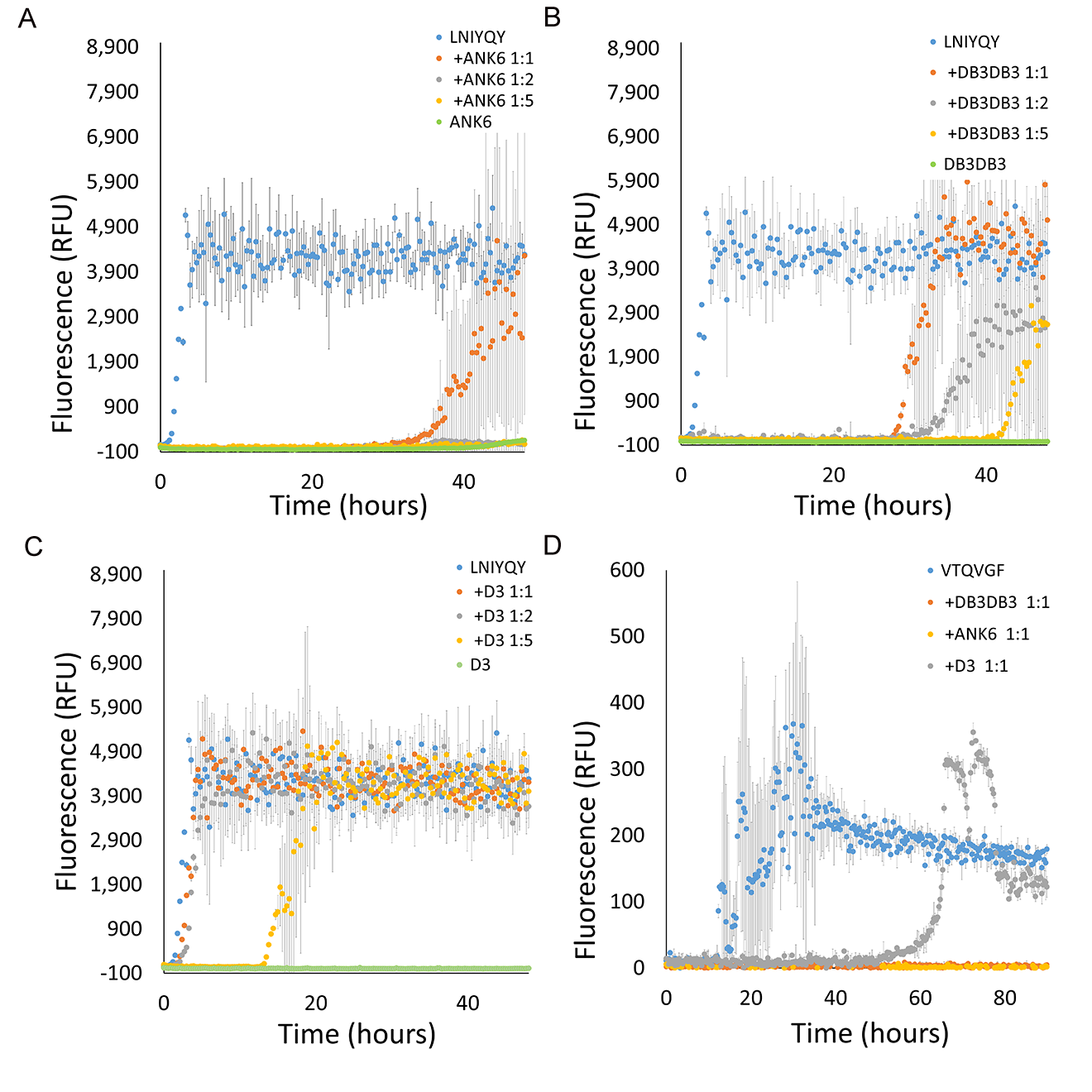

Supplement: S10 Fig — The graphs show mean fluorescence readings of triplicate ThT measurements of CsgA spine segments in the presence of the D-peptide inhibitors. Fibrillation of 100μM 45LNIYQY50 was assessed with 0, 100, 200 and 500 μM ANK6 (A), DB3DB3 (B) or D3 (C) peptides. (D) Fibrillation of 500μM 137VTQVGF142 at was examined with 500 μM of ANK6, DB3DB3 or D3 peptides. Error bars represent standard error of the mean calculated from triplicates. Each graph represents at least three independent experiments. (TIF) [file ppat.1007978.s010.tif]

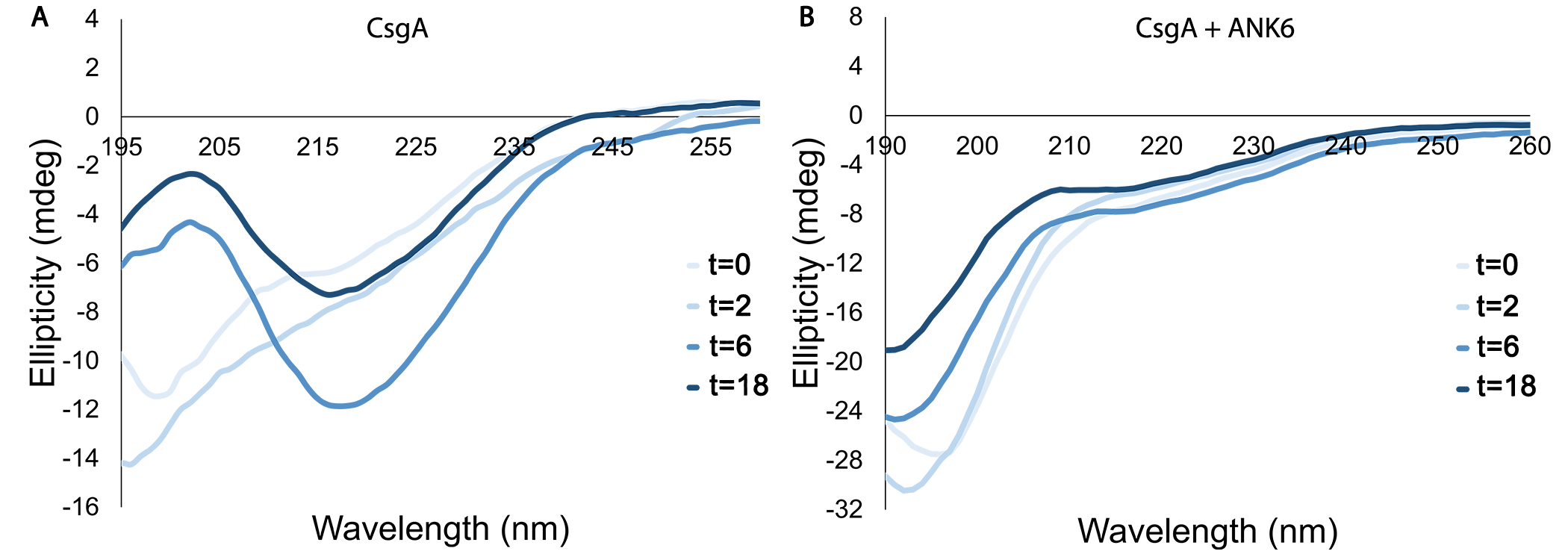

Supplement: S11 Fig — Time-dependent CD spectra of CsgA incubated alone (A) or in the presence of ANK6 (B) (1:5 molar ratio). The changes in ellipticity are shown along a wavelength range of 190–265 nm. (TIF) [file ppat.1007978.s011.tif]

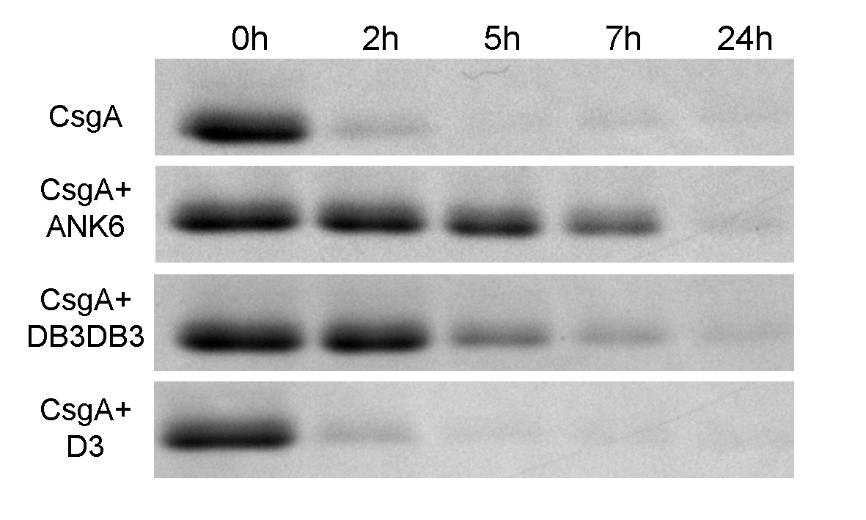

Supplement: S12 Fig — Migration of soluble CsgA detected by Coomassie-blue staining of 15% SDS-PAGE gel. Incubated CsgA forms insoluble fibrils and does not migrate on the gel compared to freshly purified CsgA. CsgA incubated with ANK6 and DB3DB3 at 1:5 molar ratios showed a prolonged soluble state of CsgA, indicating inhibition of the formation of insoluble fibrils. (TIF) [file ppat.1007978.s012.tif]

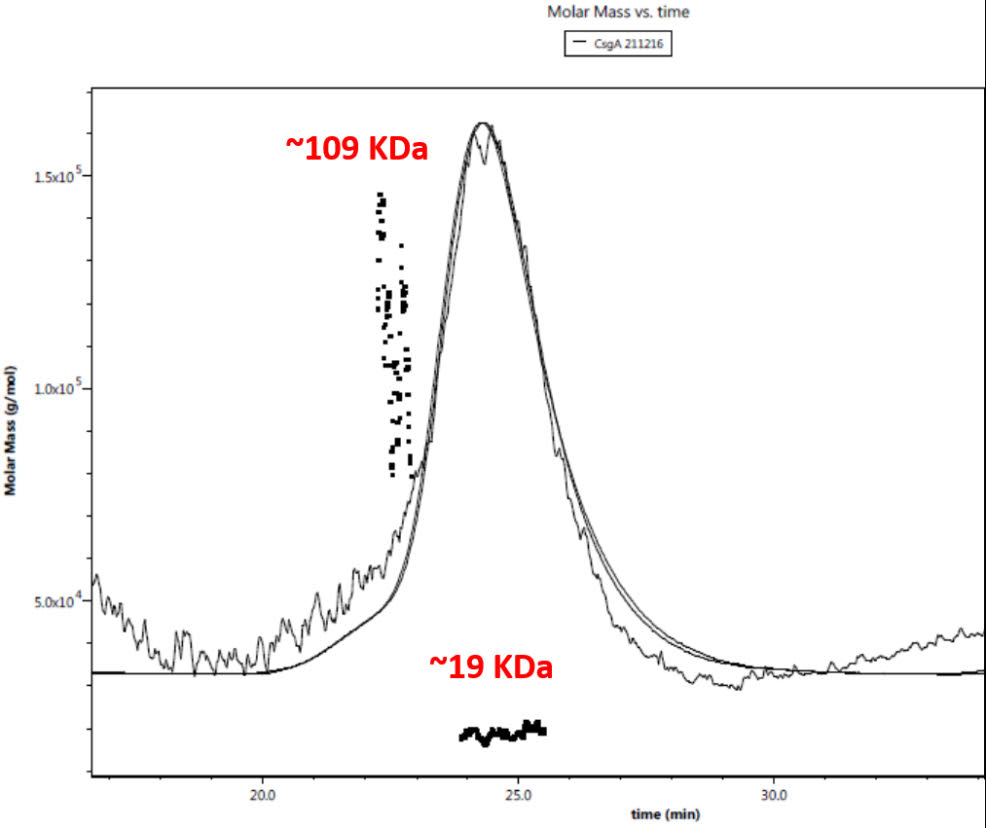

Supplement: S13 Fig — SEC-MALS chromatogram of CsgA presents two main populations with different molecular weights. The major peak corresponds to monomeric CsgA, while the minor peak corresponds to hexamers of CsgA. (TIF) [file ppat.1007978.s013.tif]

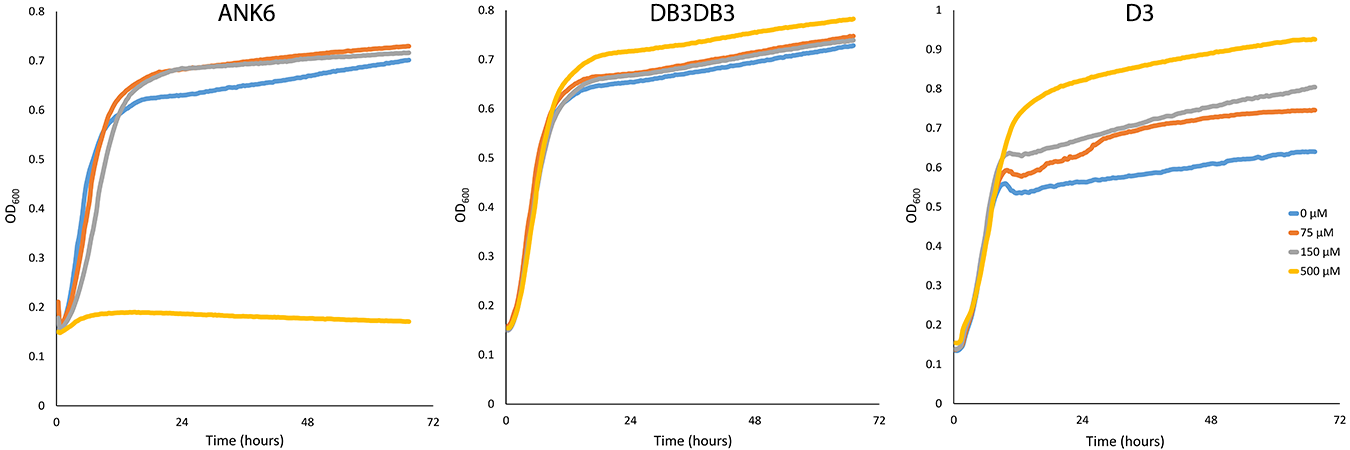

Supplement: S14 Fig — Bacterial growth of the MAE52 strain was assessed in the presence of the D-peptides at concentrations of 0, 75, 150 and 500 μM by a spectrophotometer at OD600. An average of triplicate readings was recorded every 20 minutes. No significant effect on the growth phase was evident except with 500 μM of ANK6. (TIF) [file ppat.1007978.s014.tif]

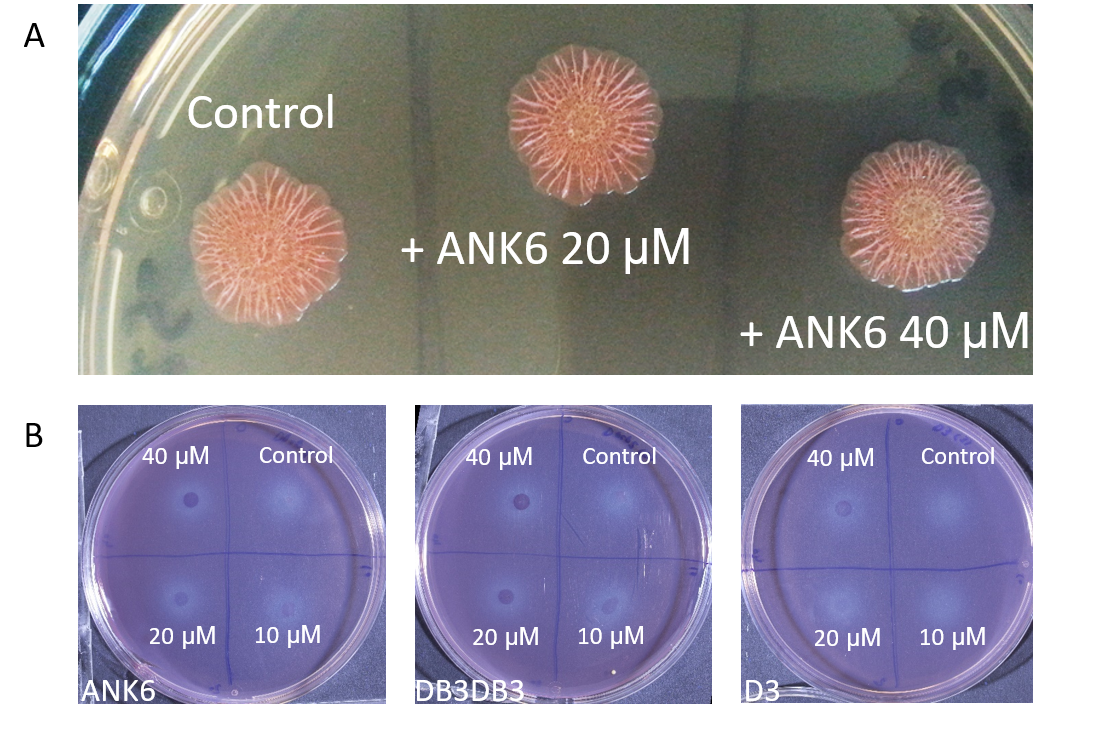

Supplement: S15 Fig — (A) S. typhimurium MAE52 strain grown on plates with CR-supplemented agar for 48 h at 30°C show reddish biofilm colony that adsorbed the dye (left colony on the image). The addition of ANK6 at 20 μM (middle colony) or 40 μM (right colony) show a dose-dependent discoloration at the center of the colony where the drop of the bacteria and D-peptide suspension was placed, indicating less CR adsorption. (B) S. typhimurium MAE150 strain (cellulose deficient mutant) colonies were grown on plates with CR-supplemented agar for 48 h at 30 °C; the images depict residual stain on the agar following the removal of the biofilm colonies. From left to right: ANK6, DB3DB3 and D3 added at different concentrations in a clockwise manner, starting in the upper-right corner (0, 10, 20 and 40 μM). Each plate represents a triplicate of repeats and the phenomenon was exhibited in three independent experiments. Quantification of the residual stain is shown in Fig 6. (TIF) [file ppat.1007978.s015.tif]
