# Supplementary material for: Structural Insights into Curli CsgA Cross-β Fibril Architecture Inspire Repurposing of Anti-amyloid Compounds as Anti-biofilm Agents
Source: PLoS Pathog. 2019 Aug 30;15(8):e1007978. doi: 10.1371/journal.ppat.1007978 (PMC6748439; doi:10.1371/journal.ppat.1007978)
Supplement: S1 Table — (DOCX) [file ppat.1007978.s016.docx]

**Table S1. Sequences of CsgA repeats and of the D-peptide inhibitors**

| **CsgA repeats** | |
| --- | --- |
| R1 | SE**LNIYQYGG**GNSALALQTDARN |
| R2 | SDLTITQHGGGNGADVGQGSDD |
| R3 | SSIDLTQRGFGNSATLDQWNGKN |
| R4 | SEMTVKQFGGGNGAAVDQTASN |
| R5 | SSVN**VTQVGF**GNNATAHQY |
| **D-peptide fibrillation inhibitors consisted of D-enantiomeric amino acids** | |
| ANK6 | RKRIRLVTKKKR-NH2 |
| DB3DB3 | RPITRLRTHQNRRPITRLRTHQNR-NH2 |
| D3 | RPRTRLHTHRNR-NH2 |

CsgA repeats are according to Wang and Chapman [34]. CsgA segments in R1 an R5 predicted to serve as amyloid spines are in bold**.**
