# Supplementary material for: Structural Insights into Curli CsgA Cross-β Fibril Architecture Inspire Repurposing of Anti-amyloid Compounds as Anti-biofilm Agents
Source: PLoS Pathog. 2019 Aug 30;15(8):e1007978. doi: 10.1371/journal.ppat.1007978 (PMC6748439; doi:10.1371/journal.ppat.1007978)
Supplement: S2 Table — (DOCX) [file ppat.1007978.s017.docx]

**Table S2. Data collection and refinement statistics (molecular replacement)**

|  | IYQYGG | LNIYQY | VTQVGF | TASNSS |
| --- | --- | --- | --- | --- |
| PDB accession code | 6G8C | 6G8D | 6G8E | 6G9G |
| Beamline | PETRA III, EMBL c/o DESY BEAMLINE P14 (MX2) | PETRA III, EMBL c/o DESY BEAMLINE P14 (MX2) | ESRF ID23-2 | PETRA III, EMBL c/o DESY BEAMLINE P14 (MX2) |
| Date | May 2, 2016 | May 2, 2016 | September 6, 2015 | May 2, 2016 |
| Crystallization conditions | 0.1 M Sodium acetate pH 4.6, 2.0 M Sodium formate | 0.1 M HEPES pH 7.5, 20%v/v Jeffamine M-600, 10 mM of TAIVVQ peptide | 3.0 M Sodium chloride, 0.1 M BIS-Tris pH 5.5 | 0.2 M Lithium sulfate, 0.1 M Tris-HCl pH 8.5, 30%(w/v) PEG 4000, 10 mM of TAIVVQ peptide |
| **Data collection** | | | | |
| Space group | C 1 2 1 | C 1 2 1 | P 1 | P 21 21 21 |
| Cell dimensions: | | | | |
| *a*, *b*, *c* (Å) | 42.17 4.76 19.60 | 42.18 4.82 26.69 | 4.81 19.34 21.90 | 9.32 12.55 21.29 |
| α, β, γ (°) | 90.0 102.4 90.0 | 90.0 126.0 90.0 | 65.8 83.7 83.2 | 90.0 90.0 90.0 |
| Number of xtals | One xtal | One xtal | Two spots from the same xtal | One xtal |
| Wavelengths (Å): | 0.97627 | 0.97627 | 0.87290 | 0.97627 |
| Resolution range (Å) | 20.6-1.65 (1.85-1.65) | 21.6-1.85  (2.1-1.85) | 19.93-1.70 (1.82-1.70) | 21.3-1.60 (1.85-1.60) |
| Total reflections | 2851 (832) | 1620 (531) | 5372 (887) | 3379 (1200) |
| Unique reflections | 545 (141) | 462 (127) | 776 (136) | 389 (130) |
| ^a^ *R*_meas_ (%) | 11.1 (35.9) | 22.5 (52.9) | 29.8 (48.1) | 23.7 (58.2) |
| *I* / sigma | 8.7 (3.2) | 3.5 (2.3) | 5.0 (3.2) | 6.1 (2.8) |
| Completeness (%) | 97.3 (100) | 96.0 (98.4) | 98.4 (99.3) | 97.7 (97.7) |
| Redundancy | 5.2 (5.9) | 3.5 (4.2) | 6.9 (6.5) | 8.7 (9.2) |
| ^b^ CC_1/2_ (%) | 99.6 (97.7) | 97.5 (89.1) | 97.9 (90.4) | 98.6 (89.0) |
| **Refinement** | | | | |
| Resolution (Å) | 19.1-1.65 (1.69-1.65) | 13.3-1.85  (1.9-1.85) | 19.9-1.70  (1.74-1.70) | 10.81-1.60 (1.79-1.60) |
| Completeness (%) | 97.3 (100) | 97.3 (100) | 100 (100) | 97.7 (100) |
| ^c^ No. reflections | 489 | 415 | 698 | 349 |
| *R*_work_ _(working set)_ (%) | 14.5 (32.0) | 17.6 (27.8) | 12.3 (17.9) | 10.4 (22.9) |
| *R*_free_ (%) | 17.8 (32.3) | 19.1 (30.7) | 13.2 (35.3) | 13.5 (23.5) |
| Test set size [%], selection | 10, random | 10, random | 10, random | 10, random |
| Number of non-hydrogen atoms: | 52 | 59 | 98 | 40 |
| Protein | 50 | 58 | Chain A: 46  Chain B: 46 | 39 |
| Ligand/ion | 0 | 0 | 0 | 0 |
| Water | 2 | 1 | 6 | 1 |
| Protein residues | 6 | 6 | 12 | 6 |
| R.m.s. deviations: | | | | |
| Bond lengths (Å) | 0.019 | 0.023 | 0.017 | 0.016 |
| Bond angles (°) | 1.67 | 1.50 | 1.53 | 1.34 |
| Average B-factor (Å^2^): | | | | |
| Average B factor for all atoms | 12.6 | 26.3 | 10.0 | 10.0 |
| Average B factor for protein | 12.1 | 25.9 | Chain A: 8.9  Chain B: 8.8 | 9.7 |
| Average B factor for water | 25.5 | 44.8 | 28.1 | 20.7 |
| Ramachandran favored (%) | 100 | 100 | 100 | 100 |
| Ramachandran outliers (%) | 0 | 0 | 0 | 0 |
| Clash score [143] | 0.00 | 0.00 | 0.00 | 0.00 |
| Molprobity score [143] | 0.50 | 0.50 | 0.50 | 0.50 |
| Molprobity percentile [143] | 100th percentile | 100th percentile | 100th percentile | 100th percentile |

Values in parenthesis are for the highest resolution shells.

^(a)^ R-meas is a redundancy-independent R-factor defined in Reference [144].

^(b)^ CC_1/2_ is percentage of correlation between intensities from random half-datasets [145].

^(c)^ Number of reflections corresponds to the working set.
