# Supplementary material for: Structural Insights into Curli CsgA Cross-β Fibril Architecture Inspire Repurposing of Anti-amyloid Compounds as Anti-biofilm Agents
Source: PLoS Pathog. 2019 Aug 30;15(8):e1007978. doi: 10.1371/journal.ppat.1007978 (PMC6748439; doi:10.1371/journal.ppat.1007978)
Supplement: S3 Table — (DOCX) [file ppat.1007978.s018.docx]

**Table S3. Features of the CsgA spine structures compared to the NNQQNY steric zipper structure**

|  | **CsgA**  **LNIYQY** | | **CsgA**  **IYQYGG** | | **CsgA**  **VTQVGF** | | **CsgA**  **TASNSS** | **Sup35**  **NNQQNY** |
| --- | --- | --- | --- | --- | --- | --- | --- | --- |
|  | Dry interface 1 ^b^ | Dry interface 2 ^b^ | Dry interface 1^b^ | Dry interface 2 ^b^ | Dry interface 1 ^b^ | Dry interface 2 ^b^ | Dry interface | Dry interface |
|  | 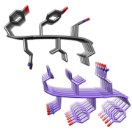 | 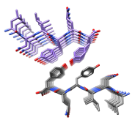 | 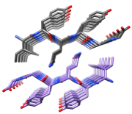 | 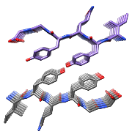 | 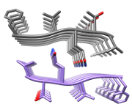 | 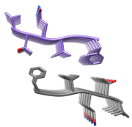 | 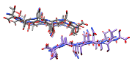 | 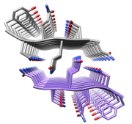 |
| **Shape complem-entarity** | 0.87 | 0.80 | 0.78 | 0.84 | 0.81 | 0.78 | 0.83 | 0.86 |
| **Inter-strand distance along the sheet** | 4.82 Å | | 4.76 Å | | 4.81 Å | | 4.66 Å (9.32 Å between antiparallel strands) | 4.87 Å |
| **Area buried of one strand within pairs of sheets ^a^** | 663 Å^2^ | 658 Å^2^ | 594 Å^2^ | 579 Å^2^ | 586 Å^2^ | 588 Å^2^ | 500 Å^2^ | 624 Å^2^ |

The values of shape complementarity, inter-strand distance and solvent exposed surface area buried calculated for the CsgA spine structures are compared with those of the NNQQNY segment from yeast prion Sup35 (PDB code 1YJO) [146] steric zipper structure. The NNQQNY was chosen for this comparison as it shows one of the highest values of shape complementarity and surface area buried among steric zipper structures [24].

^a^ The number of solvent accessible surface area buried is the average area buried of one strand within two β-sheets (total area buried from both side is double the reported number). The surface area buried was calculated with Chimera (UCSF) with default probe radius and vertex density are 1.4 Å and 2.0/Å^2^, respectively.

^b^ LNIYQY, IYQYGG, and VTQVGF display two possible dry interfaces in the crystal structure; parameters for each interface were calculated separately.
