# Supplementary material for: Structural Insights into Curli CsgA Cross-β Fibril Architecture Inspire Repurposing of Anti-amyloid Compounds as Anti-biofilm Agents
Source: PLoS Pathog. 2019 Aug 30;15(8):e1007978. doi: 10.1371/journal.ppat.1007978 (PMC6748439; doi:10.1371/journal.ppat.1007978)
Supplement: S1 References — (DOCX) [file ppat.1007978.s019.docx]

143. Chen VB, Arendall WB, 3rd, Headd JJ, Keedy DA, Immormino RM, Kapral GJ, et al. MolProbity: all-atom structure validation for macromolecular crystallography. Acta Crystallogr D Biol Crystallogr. 2010;66(Pt 1):12-21. Epub 2010/01/09. doi: 10.1107/s0907444909042073. PubMed PMID: 20057044; PubMed Central PMCID: PMCPmc2803126.

144. Diederichs K, Karplus PA. Improved R-factors for diffraction data analysis in macromolecular crystallography. Nature structural biology. 1997;4(4):269-75. Epub 1997/04/01. PubMed PMID: 9095194.

145. Karplus PA, Diederichs K. Linking crystallographic model and data quality. Science. 2012;336(6084):1030-3. Epub 2012/05/26. doi: 10.1126/science.1218231. PubMed PMID: 22628654; PubMed Central PMCID: PMCPMC3457925.

146. Nelson R, Sawaya MR, Balbirnie M, Madsen AO, Riekel C, Grothe R, et al. Structure of the cross-beta spine of amyloid-like fibrils. Nature. 2005;435(7043):773-8. Epub 2005/06/10. doi: 10.1038/nature03680. PubMed PMID: 15944695; PubMed Central PMCID: PMCPMC1479801.
